# Highly enantioselective access to cannabinoid-type tricyles by organocatalytic Diels–Alder reactions

**DOI:** 10.3762/bjoc.8.160

**Published:** 2012-08-28

**Authors:** Stefan Bräse, Nicole Volz, Franziska Gläser, Martin Nieger

**Affiliations:** 1 Karlsruhe Institute of Technology (KIT), Institute of Organic Chemistry, Fritz-Haber-Weg 6, D-76131 Karlsruhe; Tel: (+49) 721 608-48581; 2 Institute of Toxicology and Genetics, Hermann-von-Helmholtz-Platz 1, D-76344 Eggenstein-Leopoldshafen, Germany; 3 Laboratory of Inorganic Chemistry, Department of Chemistry, University of Helsinki, P.O. Box 55, FIN-00014 Helsinki, Finland

**Keywords:** cannabinoids, Diels–Alder reaction, natural product synthesis, organocatalysis

## Abstract

After prosperous domino reactions towards benzopyrans, the products were used as the starting material in Lewis acid catalyzed and organocatalytic Diels–Alder reactions to build up a tricyclic system. Herein, an asymmetric induction up to 96% enantiomeric excess was obtained by the use of imidazolidinone catalysts. This approach can be utilized to construct the tricyclic system in numerous natural products, in particular the scaffold of tetrahydrocannabinol (THC) being the most representative one. Compared with other published methods, condensation with a preexisting cyclohexane moiety in the precursor is needed to gain the heterogenic tricycle systems, whereas we present a novel strategy towards cannabinoid derivatives based on a flexible modular synthesis.

## Introduction

**Figure 1:**
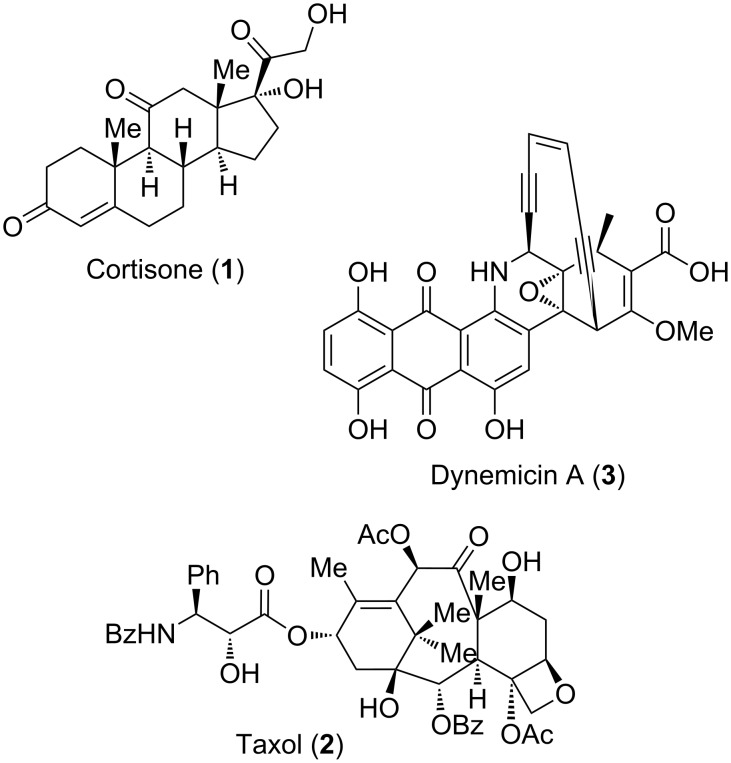
An assortment of natural products synthesized by Diels–Alder reactions.

The Diels–Alder reaction is one of the most important processes for carbon–carbon-bond formation in organic chemistry [1–2]. Especially in the synthesis of natural products it is a widely used method [3–7]. Some examples are shown in Figure 1. The first application was the total synthesis of the steroid Cortisone (**1**) in 1952 by Woodward et al. [8]. Another example, indicating the importance of the well-known [4 + 2] cycloaddition in natural-product synthesis, is the first published total synthesis of Taxol (**2**) by Nicolaou. Two different [4 + 2]-cycloaddition reactions were applied to set up each of the two six-membered rings of the target molecule [9–10]. As a final example, Dynemicin A (**3**) should be mentioned, which is an enediyne consisting of a complex heterocyclic skeleton and a network of sensitive functional groups, exhibiting antitumor and antibiotic activity [11]. Three independent research groups (Schreiber, Myers and Danishefsky) successfully applied [4 + 2]-cycloaddition reactions in elegant and divergent strategies to reach the target molecule [12–14].

Given the importance of the Diels–Alder reaction, considerable efforts have been directed towards increasing the reaction rate and enantioselectivity. In the past century, catalysts that were employed for the enantioselective synthesis of organic compounds, such as pharmaceuticals, agrochemicals, or fine chemicals, were either transition-metal complexes or enzymes. In the past few years, however, organocatalysis has emerged as an alternative approach for the catalytic production of enantiomerically pure organic compounds [15–16]. These organocatalysts have several important advantages. They are metal-free, usually nontoxic, stable, moisture-insensitive, and often easy to obtain. Because of their inertness towards oxygen and moisture, the use of absolute solvents, inert atmosphere, low temperature, etc., is, in many instances, not required. Furthermore, due to the absence of transition metals, organocatalytic methods are especially attractive for the preparation of compounds that do not tolerate metal contamination, e.g., active pharmaceutical ingredients.

MacMillan’s imidazolidinone-based organocatalysts are general catalysts for a variety of asymmetric transformations. The first highly enantioselective organocatalytic Diels–Alder reaction was reported by MacMillan in his pioneering work in 2000 [17]. The activated iminium ion, formed through condensation of imidazolidinone and an α,β-unsaturated aldehyde, underwent reactions with various dienes to yield [4 + 2]-cycloadducts in excellent yields and enantioselectivities.

## Results and Discussion

**Figure 2:**
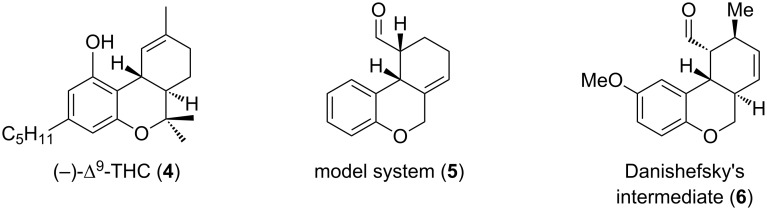
Intermediates towards the total synthesis of (−)-Δ^9^-tetrahydrocannabinol (**4**).

On our way towards a metal-free total synthesis of (−)-Δ^9^-tetrahydrocannabinol (**4**, Figure 2) we developed a convenient organocatalytic Diels–Alder route to generate the desired tricycle **5** of a model-system that also represents an alternative approach to key-intermediate **6** in Danishefsky’s total synthesis of Dynemicin A (**3**) [14].

Our strategy, using the [4 + 2]-cycloaddition to obtain the cannabinoid tricycle system, is able to employ a variety of dienophiles and, hence, provides a versatile entry to this significant group of naturally occurring compounds [18], whereas the achieved Diels–Alder products can be further modified to gain a variety of heterocycles. Most published strategies are based on the use of a preexisting cyclohexane moiety in the starting material to form the heterogenic tricycle [19–28].

**Scheme 1:**
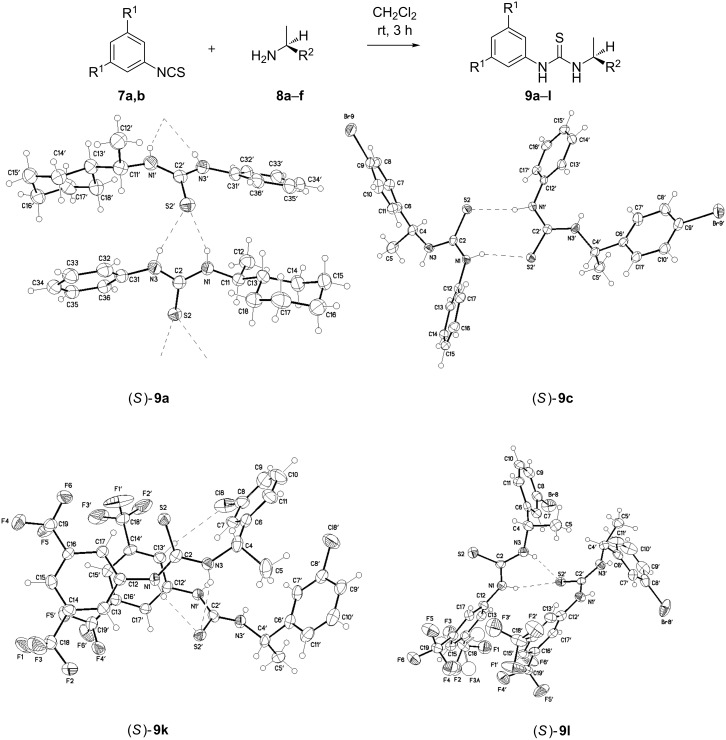
Synthesis of thiourea catalysts **9a–l**.

In former experiments of our group [29–31] it became apparent that without a catalyst no conversion towards the desired product occurred. Only the homodimer of the diene **10** could be isolated. Metal-based Lewis acids (e.g., catechol boronates) proved to be inefficient or too reactive. Due to the fact that other groups have used thiourea-derivatives as successful catalysts [32], we decided to test, on a model system, whether these catalysts could increase the reaction rate based on specific hydrogen bonds. In our initial screening we used the proven Schreiner catalyst 1,3-bis(3,5-bis(trifluoromethyl)phenyl)thiourea, which gave a satisfying 60% yield. In order to optimize the yield and to introduce chiral elements, we screened a number of analogues. These thiourea catalysts **9a**–**l** were easy to obtain from the corresponding isothiocyanates **7a**,**b** and chiral amines **8a**–**f** in a one-step synthesis (Scheme 1) [33]. The yields were good to excellent in all procedures (Table 1).

**Table 1:** Yields of thiourea syntheses.

Entry	R^1^	R^2^	Product	Yield [%]

1	H	Cy	(*R*)-**9a** [34]	99
2	H	Cy	(*S*)-**9a** [34]	95
3	H	Ph	(*R*)-**9b**	99
4	H	Ph	(*S*)-**9b** [34]	99
5	H	*p*-BrC_6_H_4_	(*R*)-**9c**	99
6	H	*p*-BrC_6_H_4_	(*S*)-**9c**	99
7	H	cyclopropyl	(*R*)-**9d**	99
8	H	*m*-ClC_6_H_4_	(*S*)-**9e**	99
9	H	*m*-BrC_6_H_4_	(*S*)-**9f**	99
10	CF_3_	Cy	(*R*)-**9g**	69
11	CF_3_	Cy	(*S*)-**9g**	62
12	CF_3_	Ph	(*R*)-**9h**	89
13	CF_3_	Ph	(*S*)-**9h** [35]	99
14	CF_3_	*p*-BrC_6_H_4_	(*R*)-**9i**	99
15	CF_3_	*p*-BrC_6_H_4_	(*S*)-**9i**	99
16	CF_3_	cyclopropyl	(*R*)-**9j**	91
17	CF_3_	*m*-ClC_6_H_4_	(*S*)-**9k**	99
18	CF_3_	*m*-BrC_6_H_4_	(*S*)-**9l**	76

**Scheme 2:**
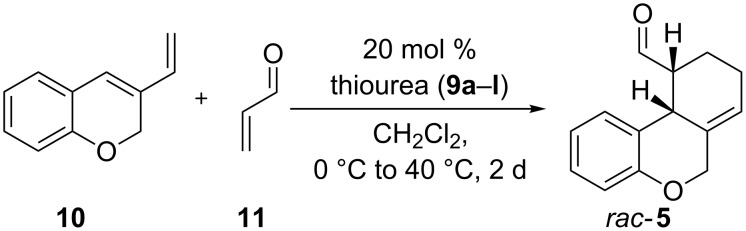
Organocatalytic Diels–Alder reaction with thiourea-catalysis.

These thioureas were used in an intermolecular Diels–Alder reaction of diene **10** [36] with acrolein (**11**) to obtain cannabinoid tricycle **5** shown in Scheme 2.

In all cases we only achieved one *cis*-diastereomer and the carbonyl function was always in the 10-position. The reaction was carried out at low temperature and high dilution to avoid the dimer formation of diene **10**. An increase of the temperature to 40 °C resulted in a higher conversion but unfortunately no enantioselectivity was observed. We obtained good to excellent yields from 68% to 99% depending on the substitution of the thiourea-catalyst **9a**–**l** (Table 2).

**Table 2:** Summarized results of the Diels–Alder reaction.

Entry	Catalyst	Yield [%]^a^

1	(*R*)-**9a**	97
2	(*S*)-**9a**	91
3	(*R*)-**9b**	99
4	(*S*)-**9b**	85
5	(*R*)-**9c**	93
6	(*S*)-**9c**	92
7	(*R*)-**9d**	74
8	(*S*)-**9e**	82
9	(*S*)-**9f**	77
10	(*R*)-**9g**	68
11	(*S*)-**9g**	78
12	(*R*)-**9h**	73
13	(*S*)-**9h**	88
14	(*R*)-**9i**	76
15	(*S*)-**9i**	85
16	(*R*)-**9j**	87
17	(*S*)-**9k**	93
18	(*S*)-**9l**	83

^a^The conversion is quantitative with respect to **10**, the byproduct is the uncatalyzed dimer of compound **10**.

When the substitution of the various thioureas **9a**–**l** is further compared with the obtained yields in the Diels–Alder reaction (Table 2), the following tendencies are also observed. A noticeable fact is that, in contrast to the thioureas with a cyclopropyl- and *m*-halophenyl-moiety (Table 2, entries 7–9), the corresponding bis(trifluoromethyl)thioureas (Table 2, entries 16–18) provide a higher conversion.

**Figure 3:**
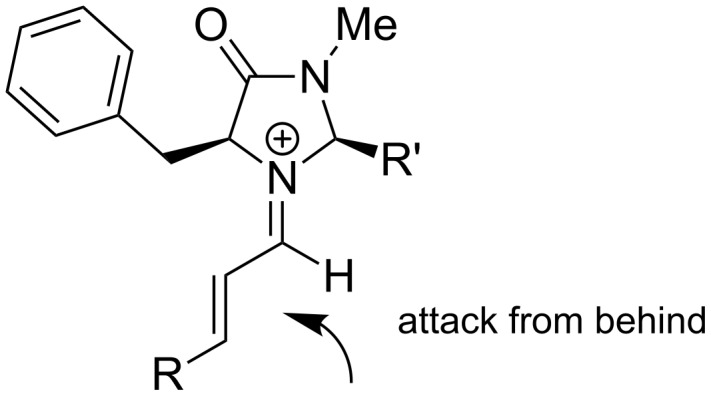
Formation of the iminium-ion.

Next to the previously described thiourea catalysts, we also analyzed iminium-ion catalysts according to MacMillan on various model systems (Figure 3) [37]. The effect of these catalysts is based on the formation of iminium ions by condensation of the dienophile bearing a carbonyl group, with the sterically hindered imidazole catalyst. In this way, one side is shielded and only the other side can be attacked by the diene. Hence, reaction rate and asymmetric induction are increased.

**Scheme 3:**
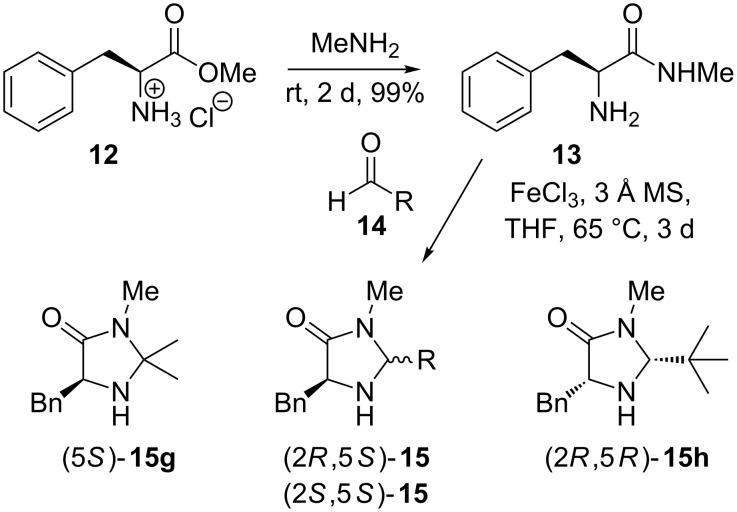
Synthesis of electron poor imidazolidinone catalysts.

The synthesis of imidazolidinone catalysts [38] is the premise for the construction of the demanding amide **13**, which was afforded by the reaction of (*S*)-phenylalanine methyl ester hydrochloride (**12**) with methylamine (Scheme 3). In the second step of the synthesis, amide **13** was cyclized with different aldehydes **14** and addition of catalytic amounts of FeCl_3_ at high temperatures into (2*S*,5*S*)- or (2*R*,5*S*)-imidazolidinone **15** [39–40].

**Figure 4:**
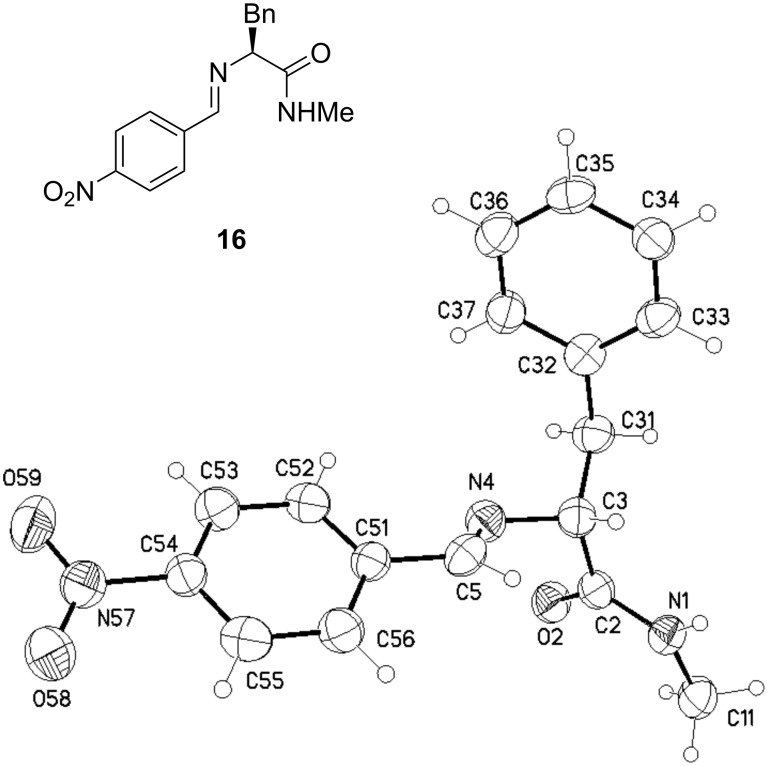
Crystal structure of the side product from the reaction of **13**.

To gain electron-poor catalysts for the degradation of the dienophilic LUMO in the Diels–Alder reaction and to consequently increase the reaction rate, we used aldehydes with electron-withdrawing groups in *ortho*-, *meta*- and *para*-positions (Scheme 3, Table 3). Reaction with *p*-nitrobenzaldehyde gave compound **16** in 66% yield (Figure 4).

**Table 3:** Results of the conversion of various aldehydes **14** with amide **13**.

Entry	(Aldehyde **14**) R =	Catalyst **15**	Yield [%]

1 2	*t*-Bu	(2*R*,5*S*)-**15a** (2*S*,5*S*)-**15a**	35 [41] 25 [40]
3 4	*o*-NO_2_-C_6_H_4_	(2*R*,5*S*)-**15b** (2*S*,5*S*)-**15b**	42 52
5 6	*m*-F-C_6_H_4_	(2*R*,5*S*)-**15c** (2*S*,5*S*)-**15c**	28 39
7 8	*p*-F-C_6_H_4_	(2*R*,5*S*)-**15d** (2*S*,5*S*)-**15d**	32 43
9 10	*p*-Br-C_6_H_4_	(2*R*,5*S*)-**15e** (2*S*,5*S*)-**15e**	39 45
11 12	*p*-CN-C_6_H_4_	(2*R*,5*S*)-**15f** (2*S*,5*S*)-**15f**	38 46

**Figure 5:**
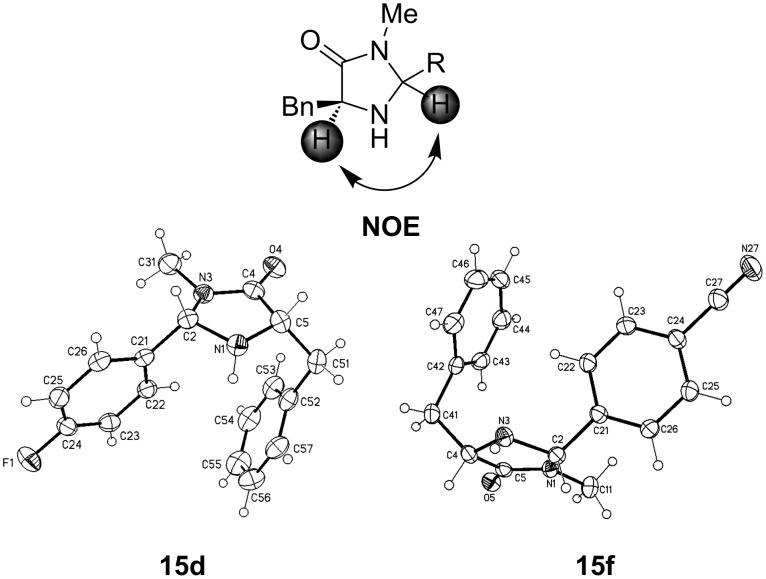
Confirmation of the relative configuration with NOESY experiments and X-ray crystal structures of two imidazolidinones **15d** and **15f**.

The organocatalytic species **15** were synthesized in moderate yields. A conspicuous feature is that the yields of the (2*S*,5*S*)-derivatives, with the exception of the *tert*-butyl substituted catalyst **15a** (Table 3, entries 1–2), are always higher than those of the corresponding (2*R*,5*S*)-components (Table 3, entries 3–12). The configuration of the afforded imidazolidinone catalysts **15** could be confirmed by NOESY experiments and from their X-ray structures (Figure 5).

**Scheme 4:**
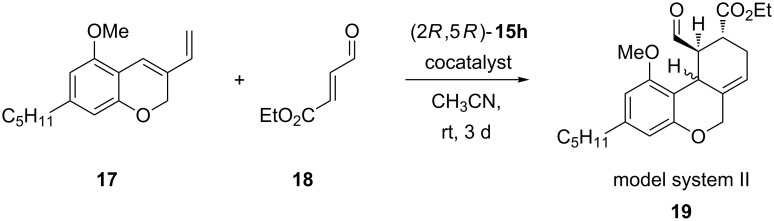
Co-catalyst screening.

Before testing the catalysts in the Diels–Alder reaction, we analyzed a few co-catalysts with the commercially available imidazolidinone catalyst **15h**. A pentyl-substituted tricycle was used as a model system (model system II) **19** (Scheme 4, Table 4).

**Table 4:** Summary of the screening of co-catalysts.

Entry	Co-catalyst	*cis*-**19**		*trans*-**19**	
		Yield [%]	ee [%]	Yield [%]	ee [%]

1	HCl (1 M)	51	96	12	91
2	HClO_4_ (60%)	6	95	10	n.d.
3	TFA	31	96	9	77
4	*p*-TSA·H_2_O	50	n.d.	15	90
5	TfOH	—	—	—	—

n.d.: not determined.

Hydrochloric acid has been proven to be the best co-catalyst (Table 4, entry 1), providing *cis*-**19** in good yield and with an enantiomeric excess of 96%. Even perchloric acid and trifluoroacetic acid gave high enantiomeric excesses but in much poorer yields (Table 4, entries 2 and 3). Using *para*-toluenesulfonic acid also afforded *cis*-**19** in good yield, but its enantiomeric excess could not be determined (Table 4, entry 4). Trifluoromethanesulfonic acid as co-catalyst only led to decomposition (Table 4, entry 5).

**Scheme 5:**
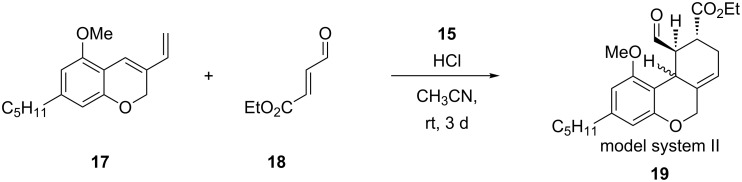
Screening of imidazolidinone catalysts **15**.

Under these optimized reaction conditions, we performed a catalyst screening with the previously synthesized imidazolidinone catalysts **15** to study their asymmetric induction (Scheme 5). As starting material we used compound **17** with the THC-typical pentyl side chain. The results are summarized in Table 5.

**Table 5:** Catalyst screening towards model system II **19**.

Entry	Catalyst **15**		*cis*-**19**	
	R =		Yield [%]	*ee* [%]

1	*t-Bu*	(2*R*,5*R*)-**15h**	51	96
2 3	*t*-Bu	(2*R*,5*S*)-**15a** (2*S*,5*S*)-**15a**	47 66	−24 −98
4 5	*o*-NO_2_-C_6_H_4_	(2*R*,5*S*)-**15b** (2*S*,5*S*)-**15b**	43 50	−89 −29
6 7	*m*-F-C_6_H_4_	(2*R*,5*S*)-**15c** (2*S*,5*S*)-**15c**	47 55	−78 −38
8 9	*p*-F-C_6_H_4_	(2*R*,5*S*)-**15d** (2*S*,5*S*)-**15d**	48 54	−79 −37
10 11	*p*-CN-C_6_H_4_	(2*R*,5*S*)-**15f** (2*S*,5*S*)-**15f**	52 54	−83 −43

The used organocatalysts, having a (*S*)-configured stereocenter in the 5-position, should prefer the formation of opposite enantiomers, compared to the commercially available (2*R*,5*R*)-**15h**. This is indicated by negative enantiomeric excess (Table 5). Due to low yields of diastereomer *trans*-**19** and the less successful determination of their enantiomeric excess, Table 5 only contains results for *cis*-**19**. Except for in the case of **15a**, we observe tendencies such as the achievement of higher yields through the use of (2*S*,5*S*)-configurated imidazolidinones **15** compared to the yields afforded by (2*R*,5*S*)-configurated catalysts **15**. There is an opposite trend for the enantiomeric excess, which is also related to the steric hindrance of the phenyl substituent, i.e., the smaller the substituent, the lower the enantiomeric excess. Application of the known *tert*-butylimidazolidinone catalyst **15a** provides the highest yield (66%) and enantiomeric excess (98% ee) with its (2*S*,5*S*)-derivate (Table 5, entry 3).

## Conclusion

In conclusion, we have demonstrated that the Diels–Alder reaction of 3-vinyl-2*H*-chromene with acrolein can be accelerated with different thioureas, obtained in a one-step synthesis (in some cases with quantitative conversion). Application of imidazolidinone catalysts, inspired by the work of MacMillan, achieved good yields up to 66% and enantiomeric excesses up to 98%.

## Experimental

### Crystal structure determinations

The single-crystal X-ray diffraction study was carried out on a Nonius Kappa-CCD (**9a**, **16**) or Bruker-Nonius APEXII diffractometer (**9c**, **9k**, **9l**, **15d**, **15f**) at 123(2) K, by using Mo Kα radiation (λ = 0.71073 Å). Direct methods (SHELXS-97) [42] were used for structure solution, and refinement was carried out with SHELXL-97 [42] (full-matrix least-squares on *F^2^*). Hydrogen atoms were localized by difference electron density determination and refined by using a riding model (H(N) free). The absolute configurations of **9a**, **9c**, **9k**, **9l** were determined by refinement of Flack’s x-parameter [43] and by using Bayesian statistics on Bijvoet differences (Hooft’s y-parameter) [44].

The absolute configuration of **15d** and **15f** could not be determined reliably by refinement of Flack’s x-parameter [43], nor by using Bayesian statistics on Bijvoet differences (Hooft’s y-parameter) [44]. The enantiomer was assigned by reference to an unchanging chiral center in the synthetic procedure.

Semi-empirical absorption corrections were applied for **9c** and **9l**. In **9l** one CF_3_-group is disordered.

**9a**: colorless, C_15_H_22_N_2_S, *M* = 262.41, crystal size 0.30 × 0.10 × 0.05 mm, monoclinic, space group *P*2_1_ (no. 4): *a* = 8.5864(5) Å, *b* = 22.2641(17) Å, *c* = 8.6811(6) Å, β = 117.350(4)°, *V* = 1474.04(17) Å^3^, *Z* = 4, ρ(calc) = 1.182 Mg m^−3^, *F*(000) = 568, μ = 0.206 mm^−1^, 8575 reflections (2θ_max_ = 50°), 4687 unique [R_int_ = 0.057], 337 parameters, 5 restraints, *R*1 (*I* > 2σ(*I*)) = 0.048, *wR2* (all data) = 0.119, GOOF = 1.09, largest diff. peak and hole 0.235/−0.290 e Å^−3^, *x* = −0.01(8), *y* = −0.05(5).

**9c**: colorless, C_15_H_15_BrN_2_S, *M* = 335.26, crystal size 0.25 × 0.10 × 0.05 mm, monoclinic, space group *P*2_1_ (no. 4): *a* = 16.2276(4) Å, *b* = 5.5562(2) Å, *c* = 17.3953(6) Å, β = 108.894(2)°, *V* = 1483.92(8) Å^3^, *Z* = 4, ρ(calc) = 1.501 Mg m^−3^, *F*(000) = 680, μ = 2.899 mm^−1^, 11045 reflections (2θ_max_ = 50°), 4958 unique [R_int_ = 0.037], 355 parameters, 5 restraints, *R*1 (*I* > 2σ(*I*)) = 0.030, *wR2* (all data) = 0.072, GOOF = 1.06, largest diff. peak and hole 0.387/−0.504 e Å^−3^, *x* = −0.018(7), *y* = −0.012(2).

**9k**: colorless, C_17_H_13_ClF_6_N_2_S, *M* = 426.80, crystal size 0.20 × 0.10 × 0.05 mm, monoclinic, space group *P*2_1_ (no. 4): *a* = 8.1359(6) Å, *b* =16.2464(13) Å, *c* = 13.9980(7) Å, β = 92.246(4)°, *V* = 1848.8(2) Å^3^, *Z* = 4, ρ(calc) = 1.533 Mg m^−3^, *F*(000) = 864, μ = 0.381 mm^−1^, 9231 reflections (2θ_max_ = 50°), 5469 unique [R_int_ = 0.039], 499 parameters, 5 restraints, *R*1 (*I* > 2σ(*I*)) = 0.055, *wR2* (all data) = 0.116, GOOF = 1.16, largest diff. peak and hole 0.388/−0.252 e Å^−3^, *x* = 0.07(9), *y* = 0.07(4).

**9l**: colorless, C_17_H_13_BrF_6_N_2_S, *M* = 471.26, crystal size 0.35 × 0.20 × 0.10 mm, monoclinic, space group *P*2_1_ (no. 4): *a* = 8.2910(7) Å, *b* = 16.1565(8) Å, *c* = 14.0204(10) Å, β = 92.073(3)°, *V* = 1876.9(2) Å^3^, *Z* = 4, ρ(calc) = 1.668 Mg m^−3^, *F*(000) = 936, μ = 2.362 mm^−1^, 10315 reflections (2θ_max_ = 50°), 5936 unique [R_int_ = 0.046], 494 parameters, 320 restraints, *R*1 (*I* > 2σ(*I*)) = 0.056, *wR2* (all data) = 0.125, GOOF = 1.09, largest diff. peak and hole 1.033/−0.634 e Å^−3^, *x* = −0.014(11), *y* = −0.009(6).

**15d**: colorless, C_17_H_17_FN_2_O, *M* = 284.33, crystal size 0.45 × 0.25 × 0.10 mm, orthorhombic, space group *P*2_1_2_1_2_1_ (no. 19): *a* = 8.4559(3) Å, *b* = 10.5000(4) Å, *c* = 16.6177(5) Å, *V* = 1475.43(9) Å^3^, *Z* = 4, ρ(calc) = 1.280 Mg m^−3^, *F*(000) = 600, μ = 0.089 mm^−1^, 11771 reflections (2θ_max_ = 50°), 2602 unique [R_int_ = 0.045], 194 parameters, 1 restraint, *R*1 (*I* > 2σ(*I*)) = 0.038, *wR2* (all data) = 0.084, GOOF = 1.11, largest diff. peak and hole 0.135/−0.173 e Å^−3^, *x* = −0.8(10), *y* = 0.3(5).

**15f**: colorless, C_18_H_17_N_3_O, *M* = 291.35, crystal size 0.50 × 0.40 × 0.30 mm, monoclinic, space group *P*2_1_ (no. 4): *a* = 6.0140(1) Å, *b* =15.7002(4) Å, *c* = 8.5232(2) Å, β = 95.211(2)°, *V* = 801.44(3) Å^3^, *Z* = 2, ρ(calc) = 1.207 Mg m^−3^, *F*(000) = 308, μ = 0.077 mm^−1^, 13805 reflections (2θ_max_ = 55°), 3628 unique [R_int_ = 0.027], 203 parameters, 2 restraints, *R*1 (*I* > 2σ(*I*)) = 0.033, *wR2* (all data) = 0.077, GOOF = 1.08, largest diff. peak and hole 0.181/−0.152 e Å^−3^, *x* = −0.2(11), *y* = 0.8(5).

**16**: colorless, C_17_H_17_N_3_O_3_, *M* = 311.34, crystal size 0.30 × 0.05 × 0.05 mm, monoclinic, space group *P*2_1_/c (no. 14): *a* = 18.265(5) Å, *b* =10.935(2) Å, *c* = 7.857(2) Å, β = 101.60(1)°, *V* = 1537.2(6) Å^3^, *Z* = 4, ρ(calc) = 1.345 Mg m^−3^, *F*(000) = 656, μ = 0.094 mm^−1^, 4930 reflections (2θ_max_ = 50°), 2685 unique [R_int_ = 0.128], 212 parameters, 1 restraint, *R*1 (*I* > 2σ(*I*)) = 0.071, *wR2* (all data) = 0.185, GOOF = 0.99, largest diff. peak and hole 0.227/−0.247 e Å^−3^.

Crystallographic data (excluding structure factors) for the structures reported in this work have been deposited with the Cambridge Crystallographic Data Centre as supplementary publications no.’s CCDC-865479 (**9a**), CCDC-865480 (**9c**), CCDC-865481 (**9k**), CCDC-865482 (**9l**), CCDC-965483 (**15d**), CCDC-865484 (**15f**), and CCDC-865485 (**16**). Copies of the data can be obtained free of charge on application to The Director, CCDC, 12 Union Road, Cambridge DB2 1EZ, UK (Fax: int. code +(1223)336-033; email: deposit@ccdc.cam.ac.uk).

## Supporting Information

Supporting Information File:

File 1 Experimental data for all new compounds.
