# Supplementary material for: Highly enantioselective access to cannabinoid-type tricyles by organocatalytic Diels–Alder reactions
Source: Beilstein J Org Chem. 2012 Aug 28;8:1385–92. doi: 10.3762/bjoc.8.160 (PMC3458765; doi:10.3762/bjoc.8.160)
Supplement: File 1 — Experimental data for all new compounds. [file Beilstein_J_Org_Chem-08-1385-s001.pdf]

Supporting Information

for

Highly enantioselective access to cannabinoid-type tricyles by organocatalytic Diels–Alder reactions

Stefan Bräse*[1,2], Nicole Volz[1], Franziska Gläser[1] and Martin Nieger[3]

Address: [1]Karlsruhe Institute of Technology (KIT), Institute of Organic Chemistry, Fritz-Haber-Weg 6, D-76131 Karlsruhe; Tel: (+49) 721 608-48581, [2]Institute of Toxicology and Genetics, Hermann-von-Helmholtz-Platz 1, D-76344 Eggenstein-Leopoldshafen, Germany and [3]Laboratory of Inorganic Chemistry, Department of Chemistry, University of Helsinki, P.O. Box 55, FIN-00014 Helsinki, Finland

Email: Stefan Bräse* - braese@kit.edu

* Corresponding author

Experimental data for all new compounds

General: All reactions were carried out in flame-dried glassware under argon. The solvents were purified by distillation over the drying solvents indicated and were transferred under argon. THF (Na/K), CH_2Cl_2 ($CaCl_2$). IR: Bruker IFS 88 spectrometer, wavenumbers in cm^{-1}. MS (EI): Finnigan MAT 95. NMR: Spectra were recorded on a Bruker Avance 400 or Avance DRX 500 spectrometer in the solvents indicated; ^{1}H and ^{13}C chemical shifts (δ) are given in ppm relative to TMS, coupling constants (*J*) in Hz. The solvent signals were used as references and the chemical shifts were converted to the TMS scale. Melting points: Laboratory Devices Inc., model Mel-Temp II; elemental analyses: elementar (model vario MICRO CUBE). Unless stated otherwise, all commercially available compounds (Acros, Fluka, Aldrich) were used as received.

***cis*-8,9,10,10a-Tetrahydro-6*H*-benzo[*c*]chromene-10-carbaldehyde (*cis*-5):**

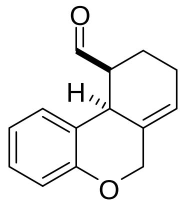

Acrolein (**11**, 235 µL, 197 mg, 3.16 mmol, 5.00 equiv) was added to a solution of 1,3-bis(3,5-bis(trifluoromethyl)phenyl)thiourea (31.6 mg, 63.2 µmol, 0.10 equiv) in methanol/water (4.3 mL/0.4 mL). The mixture was stirred for 2 min at rt, before 3-vinyl-2*H*-chromene (100 mg, 632 µmol, 1.00 equiv), dissolved in methanol (2 mL), was added at 0 °C over a period of 20 min. The resulting mixture was stirred for 2 d at 40 °C. For workup, the reaction mixture was diluted with Et_2O (5 mL), and the organic phase was washed with brine (10 mL) and dried over sodium sulfate. The solvent was removed under reduced pressure and the product was purified by flash column chromatography (*n*-Hex/EtOAc 80:1).

75.8 mg (56%). – R_f (*n*-Hex/EtOAc 1:1) = 0.40. – ^{1}H NMR (500 MHz, $CDCl_3$): δ/ppm = 1.88 (ddt, ^2J = 11.4 Hz, ^3J = 3.5 Hz, ^3J = 1.1 Hz, 1 H, 9-*H*), 2.15–2.19 (m, 2 H, 8-*H*), 2.36 (ddt, ^2J = 11.4 Hz, ^3J = 6.2 Hz, ^3J = 3.5 Hz, 1 H, 9-*H*), 3.11 (dtd, ^3J = 6.8 Hz, ^3J = 3.5 Hz, ^3J = 1.6 Hz, 1 H, 10-*H*), 3.95 (d, ^3J = 6.8 Hz, ^4J = 3.5 Hz, 1 H, 10a-*H*), 4.50 (s, 2 H, 6-*H*), 5.87 (q, ^3J = 3.7 Hz, 1 H, 7-*H*), 6.88 (dd, ^3J = 8.1 Hz, ^4J = 0.7 Hz, 1 H, 4-H_{ar}), 6.95 (dd, ^3J = 8.1 Hz, ^3J = 7.6 Hz, 1 H, 2-H_{ar}), 7.13 (dd, ^3J = 8.1 Hz, ^3J = 7.6 Hz, 1 H, 3-H_{ar}), 7.19 (d, ^3J = 7.6 Hz, 1 H, 1-H_{ar}), 9.30 (dd, ^3J = ^3J = 1.6 Hz, 1 H, C*H*O). – ^{13}C NMR (125 MHz, $CDCl_3$): δ/ppm = 21.5 (s, *C*-8), 23.6 (s, *C*-9), 35.6 (t, *C*-10a), 48.6 (t, *C*-10), 70.2 (s, *C*-6), 117.6 (t, C_{ar}-4), 121.6 (t, C_{ar}-2), 123.0 (q, C_{ar}-10b), 126.1 (t, *C*-7), 127.7 (t, C_{ar}-3), 127.9 (t, C_{ar}-1), 131.1 (q, *C*-6a), 155.9 (q, C_{ar}-4a), 204.7 (t, *C*HO). – IR (KBr): ṽ/cm^{-1} = 2926 (w), 2841 (w),

1718 (s), 1606 (w), 1580 (w), 1487 (s), 1458 (m), 1376 (w), 1306 (m), 1278 (w), 1240 (m), 1220 (m), 1182 (w), 1118 (w), 1039 (m), 998 (m), 940 (w), 757 (m), 723 (w). – EI-MS (70 eV) *m/z* (%): 214 (100) [M^+], 185 (30) [$M^+{-}CO$], 158 (78) [$M^+{-}C_3H_4O$], 131 (30) [$M^+{-}C_5H_7O$]. – EI-HRMS ($C_{14}H_{14}O_2$): calcd. 214.0992, found 214.0994.

General procedure for the preparation of thiourea-catalysts 9

Substituted amine **8** (1.00 equiv) was added to a solution of isothiocyanate **7** (1.00 equiv) in CH_2Cl_2 (2 mL/mmol amine). The resulting mixture was stirred for 3 h at rt. The solvent was removed under reduced pressure and the product was purified by flash column chromatography.

(*R*)-1-(1-Cyclohexylethyl)-3-phenylthiourea ((*R*)-9a) [1]:

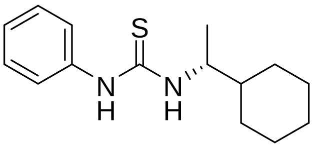

377 mg, 99% (scale: 1.45 mmol). – R_f (*n*-Hex/EtOAc 10:1) = 0.20. – mp: 119–121 °C. – $[\alpha]_D^{20}$ = −68.0 (*c* = 1.05, $CHCl_3$). – ^1H NMR (400 MHz, $CDCl_3$): δ/ppm = 0.86–1.27 (m, 5 H, CH_2), 1.10 (d, ^3J = 6.7 Hz, 3 H, CH_3), 1.37–1.46 (m, 1 H, C*H*), 1.57–1.74 (m, 5 H, CH_2), 4.40 (dd, ^3J = 8.3 Hz, ^3J = 6.7 Hz, 1 H, NHC*H*), 5.87 (d, ^3J = 8.3 Hz, 1 H, N*H*), 7.18–7.31 (m, 2 H, 2 × H_{ar}), 7.27–7.31 (m, 1 H, H_{ar}), 7.41–7.45 (m, 2 H, 2 × H_{ar}), 8.03 (s, 1 H, Ar-N*H*). – ^{13}C NMR (100 MHz, $CDCl_3$): δ/ppm = 17.2 (p, CH_3), 26.0 (s, CH_2), 26.1 (s, CH_2), 26.3 (s, CH_2), 28.9 (s, CH_2), 29.2 (s, CH_2), 42.8 (t, *C*H), 55.7 (t, NH*C*H), 125.1 (t, C_{ar}H), 127.1 (t, 2 × C_{ar}H), 130.2 (t, 2 × C_{ar}H), 136.1 (q, C_{ar}), 179.6 (q, *C*=S). – IR (DRIFT): ṽ/cm^{-1} = 3236 (m), 3064 (m), 2922 (m), 2852 (m), 1599 (m), 1551 (s), 1497 (m), 1449 (m), 1352 (m), 1314 (m), 1260 (m), 1207 (m), 1155 (m), 1074 (w), 1028 (w), 998 (w), 962 (w), 903 (w), 892 (w), 759 (m), 700 (m), 606 (m), 493 (w). – EI-MS *m/z* (%): 262 (100) [M^+], 153 (54), 94 (28). – EI-HRMS ($C_{15}H_{22}N_2S$): calcd. 262.1504, found 262.1507. – $C_{15}H_{22}N_2S$ (262.2): calcd. C 68.66, H 8.45, N 10.68, S 12.22, found C 68.71, H 8.02, N 10.80, S 12.39.

(*S*)-1-(1-Cyclohexylethyl)-3-phenylthiourea ((*S*)-9a) [1]:

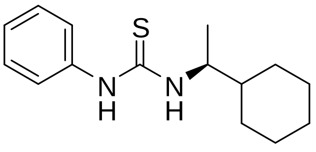

360 mg, 95% (scale: 1.45 mmol). – R_f (*n*-Hex/EtOAc 5:1) = 0.30. – mp: 118–121 °C. – $[\alpha]_D^{20}$ = −77.6 (*c* = 0.99, $CHCl_3$). – ^1H NMR (400 MHz, $CDCl_3$): δ/ppm = 0.86–1.24 (m, 5 H, CH_2), 1.10 (d, ^3J = 6.7 Hz, 3 H, CH_3), 1.37–1.46 (m, 1 H, C*H*), 1.57–1.74 (m, 5 H, CH_2), 4.39–4.41 (m, 1 H, NHC*H*), 5.87 (d, ^3J = 8.2 Hz, 1 H, N*H*), 7.18–7.20 (m,

2 H, 2 × H_{ar}), 7.27–7.30 (m, 1 H, H_{ar}), 7.40–7.44 (m, 2 H, 2 × H_{ar}), 8.05 (s, 1 H, Ar-N*H*). – ^{13}C NMR (100 MHz, $CDCl_3$): δ/ppm = 17.2 (p, CH_3), 26.0 (s, CH_2), 26.1 (s, CH_2), 26.3 (s, CH_2), 28.9 (s, CH_2), 29.2 (s, CH_2), 42.8 (t, *C*H), 55.6 (t, NH*C*H), 125.1 (t, C_{ar}H), 127.1 (t, 2 × C_{ar}H), 130.2 (t, 2 × C_{ar}H), 136.1 (q, C_{ar}), 179.6 (q, *C*=S). – IR (DRIFT): ṽ/cm^{-1} = 3234 (s), 3067 (s), 2930 (s), 2854 (s), 2671 (w), 1967 (w), 1947 (w), 1864 (w), 1795 (w), 1736 (w), 1602 (m), 1552 (s), 1497 (m), 1450 (m), 1352 (s), 1314 (s), 1260 (m), 1208 (m), 1155 (m), 1134 (m), 1111 (m), 1074 (m), 1046 (m), 1028 (m), 998 (m), 962 (m), 903 (m), 892 (m), 849 (w), 836 (m), 794 (w), 759 (m), 701 (s), 606 (m), 530 (w), 496 (m), 453 (w). – EI-MS *m/z* (%): 262 (100) [M^+], 153 (83) [$C_7H_9N_2S^+$], 151 (45) [$C_7H_7N_2S^+$], 136 (42) [$C_7H_6NS^+$], 93 (58) [$C_6H_7N^+$], 77 (53) [$C_6H_5^+$], 55 (85), 41 (57). – EI-HRMS ($C_{15}H_{22}N_2S$): calcd. 262.1504, found 262.1502. – $C_{15}H_{22}N_2S$ (262.2): calcd. C 68.66, H 8.45, N 10.68, S 12.22, found C 68.61, H 7.79, N 10.89, S 12.81.

(*R*)-1-Phenyl-3-(1-phenylethyl)thiourea ((*R*)-9b) [2]:

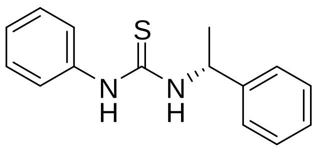

365 mg, 99% (scale: 1.45 mmol). – R_f (*n*-Hex/EtOAc 4:1) = 0.30. – mp: 62–66 °C. – $[\alpha]_D^{20}$ = −94.4 (*c* = 0.98, $CHCl_3$). – ^{1}H NMR (400 MHz, $CDCl_3$): δ/ppm = 1.46 (d, ^3J = 6.9 Hz, 3 H, CH_3), 5.60 (bs, 1 H, NHC*H*), 6.19 (bs, 1 H, N*H*), 7.09–7.19 (m, 2 H, 2 × H_{ar}), 7.17–7.28 (m, 6 H, 6 × H_{ar}), 7.30–7.34 (m, 2 H, 2 × H_{ar}), 7.93 (bs, 1 H, Ar-N*H*). – ^{13}C NMR (100 MHz, $CDCl_3$): δ/ppm = 21.5 (p, CH_3), 54.3 (t, *C*H), 125.0 (t, 2 × C_{ar}H), 126.1 (t, 2 × C_{ar}H), 127.2 (t, C_{ar}H), 127.5 (t, C_{ar}H), 128.7 (t, 2 × C_{ar}H), 130.1 (t, 2 × C_{ar}H), 136.1 (q, C_{ar}), 142.1 (q, C_{ar}), 179.7 (q, *C*=S). – IR (DRIFT): ṽ/cm^{-1} = 3338 (s), 3084 (m), 3058 (m), 3027 (m), 2980 (m), 2924 (m), 2866 (m), 2336 (w), 1953 (w), 1881 (w), 1809 (w), 1758 (w), 1591 (s), 1535 (s), 1496 (s), 1455 (s), 1385 (s), 1357 (m), 1312 (s), 1238 (s), 1200 (s), 1141 (m), 1085 (m), 1024 (m), 959 (m), 942 (m), 913 (m), 845 (m), 808 (w), 761 (s), 745 (m), 698 (s), 643 (m), 603 (m), 521 (s), 409 (w). – EI-MS *m/z* (%): 256 (59) [M^+], 118 (29), 105 (100) [$C_8H_9^+$], 77 (50) [$C_6H_5^+$], 57 (22) [CNS^+]. – EI-HRMS ($C_{15}H_{16}N_2S$): calcd. 256.1034, found 256.1034.

(*S*)-1-Phenyl-3-(1-phenylethyl)thiourea ((*S*)-9b) [3]:

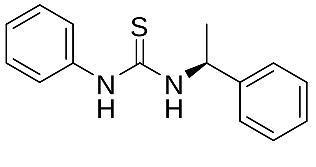

378 mg, 99% (scale: 1.45 mmol). – R_f (*n*-Hex/EtOAc 5:1) = 0.20. – mp: 59–64 °C. – $[\alpha]_D^{20}$ = −90.0 (*c* = 1.08,

$CHCl_3$). – ^{1}H NMR (400 MHz, $CDCl_3$): δ/ppm = 1.54 (d, ^3J = 6.9 Hz, 3 H, CH_3), 5.68 (s, 1 H, C*H*), 6.28 (s, 1 H, N*H*), 7.17–7.19 (m, 2 H, 2 × H_{ar}), 7.24–7.35 (m, 6 H, 6 × H_{ar}), 7.38–7.41 (m, 2 H, 2 × H_{ar}), 8.07 (s, 1 H, Ar-N*H*). – ^{13}C NMR (100 MHz, $CDCl_3$): δ/ppm = 21.5 (p, CH_3), 54.3 (t, *C*H), 125.0 (t, C_{ar}H), 126.1 (t, C_{ar}H), 127.1 (t, 2 × C_{ar}H), 127.5 (t, 2 × C_{ar}H), 128.7 (t, 2 × C_{ar}H), 130.1 (t, 2 × C_{ar}H), 136.1 (q, C_{ar}), 142.1 (q, C_{ar}), 179.7 (q, *C*=S). – IR (DRIFT): ṽ/cm^{-1} = 3338 (s), 3028 (m), 2981 (m), 2924 (m), 2867 (m), 2336 (m), 1954 (w), 1881 (w), 1181 (w), 1758 (w), 1596 (s), 1536 (s), 1496 (s), 1455 (s), 1386 (m), 1357 (m), 1312 (s), 1238 (s), 1200 (s), 1141 (m), 1085 (m), 1024 (m), 959 (m), 942 (m), 913 (m), 845 (w), 809 (m), 761 (s), 699 (s), 643 (m), 604 (m), 521 (m), 409 (w). – EI-MS *m/z* (%): 256 (12) [M^+], 151 (24) [$C_7H_7N_2S^+$], 120 (27) [$C_8H_{10}N^+$], 105 (100) [$C_8H_9^+$], 93 (52) [$C_6H_7N^+$], 77 (67) [$C_6H_5^+$]. – EI-HRMS ($C_{15}H_{16}N_2S$): calcd. 256.1034, found 256.1031. – $C_{15}H_{16}N_2S$ (256.1): calcd. C 70.27, H 6.29, N 10.93, S 12.51, found C 70.03, H 5.82, N 11.00, S 13.27.

(*R*)-1-(1-(4-Bromophenyl)ethyl)-3-phenylthiourea ((*R*)-9c):

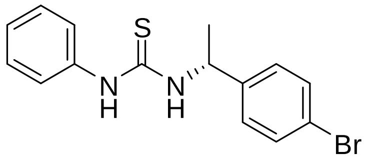

489 mg, 99% (scale: 1.45 mmol). – R_f (*n*-Hex/EtOAc 5:1) = 0.20. – mp: 128–131 °C. – $[\alpha]_D^{20}$ = −128 (*c* = 1.02, $CHCl_3$). – ^{1}H NMR (400 MHz, $CDCl_3$): δ/ppm = 1.49 (d, ^3J = 6.9 Hz, 3 H, CH_3), 5.62–5.66 (m, 1 H, C*H*), 6.18 (d, ^3J = 4.7 Hz, 1 H, N*H*), 7.16–7.18 (m, 4 H, 4 × H_{ar}), 7.28–7.31 (m, 1 H, H_{ar}), 7.40–7.45 (m, 4 H, 4 × H_{ar}), 8.07 (s, 1 H, Ar-N*H*). – ^{13}C NMR (100 MHz, $CDCl_3$): δ/ppm = 21.4 (p, CH_3), 53.7 (t, *C*H), 121.3 (q, C_{ar}-Br), 125.1 (t, C_{ar}H), 127.4 (t, 2 × C_{ar}H), 127.9 (t, 2 × C_{ar}H), 130.3 (t, 2 × C_{ar}H), 131.8 (t, 2 × C_{ar}H), 135.9 (q, C_{ar}), 141.4 (q, C_{ar}), 179.8 (q, *C*=S). – IR (DRIFT): ṽ/cm^{-1} = 3391 (m), 3204 (m), 2972 (m), 2924 (w), 2869 (w), 1956 (w), 1896 (w), 1754 (w), 1590 (m), 1541 (m), 1503 (m), 1448 (m), 1406 (m), 1379 (m), 1313 (m), 1244 (m), 1200 (m), 1176 (m), 1145 (m), 1101 (m), 1073 (m), 1028 (m), 1008 (m), 946 (w), 914 (w), 835 (w), 917 (m), 785 (w), 748 (m), 714 (m), 695 (m), 645 (m), 611 (m), 513 (m), 490 (m), 458 (w). – EI-MS *m/z* (%): 336/334 (100/98) [M^+], 200/198 (25/25) [$C_8H_{10}BrN^+$], 185/183 (51/50) [$C_8H_9Br^+$], 151 (30) [$C_7H_7N_2S^+$], 105 (38) [$C_8H_9^+$], 93 (75) [$C_6H_7N^+$], 77 (44) [$C_6H_5^+$]. – EI-HRMS ($C_{15}H_{15}BrN_2S$): calcd. 334.0139, found 334.0136. – $C_{15}H_{15}BrN_2S$ (334.0): calcd. C 53.74, H 4.51, N 8.36, S 9.56, found C 53.68, H 4.33, N 8.39, S 9.54.

(*S*)-1-(1-(4-Bromophenyl)ethyl)-3-phenylthiourea ((*S*)-9c):

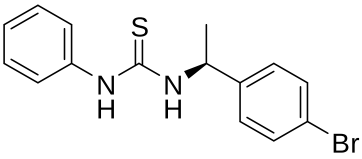

481 mg, 99% (scale: 1.45 mmol). – R_f (*n*-Hex/EtOAc 5:1) = 0.20. – mp: 126–129 °C. – $[\alpha]_D^{20}$ = −125 (*c* = 0.98, $CHCl_3$). – ^1H NMR (400 MHz, $CDCl_3$): δ/ppm = 1.49 (d, ^3J = 6.9 Hz, 3 H, CH_3), 5.62–5.66 (m, 1 H, C*H*), 6.19 (d, ^3J = 3.7 Hz, 1 H, N*H*), 7.16–7.18 (m, 4 H, 4 × H_{ar}), 7.27–7.31 (m, 1 H, H_{ar}), 7.39–7.45 (m, 4 H, 4 × H_{ar}), 8.18 (s, 1 H, Ar-N*H*). – ^{13}C NMR (100 MHz, $CDCl_3$): δ/ppm = 21.4 (p, CH_3), 53.7 (t, *C*H), 121.3 (q, C_{ar}-Br), 125.1 (t, C_{ar}H), 127.3 (t, 2 × C_{ar}H), 127.9 (t, 2 × C_{ar}H), 130.2 (t, 2 × C_{ar}H), 131.7 (t, 2 × C_{ar}H), 135.9 (q, C_{ar}), 141.4 (q, C_{ar}), 179.8 (q, *C*=S). – IR (DRIFT): ṽ/cm^{-1} = 3390 (s), 3205 (s), 2972 (s), 2923 (m), 2868 (m), 1956 (w), 2795 (w), 1753 (w), 1590 (s), 1542 (s), 1504 (s), 1448 (s), 1406 (m), 1381 (s), 1313 (s), 1244 (s), 1200 (s), 1175 (m), 1145 (s), 1101 (s), 1073 (s), 1028 (m), 1008 (s), 946 (m), 914 (m), 835 (m), 817 (s), 785 (m), 748 (s), 714 (s), 695 (s), 645 (s), 611 (s), 513 (s), 490 (s), 459 (s), 409 (w). – EI-MS *m/z* (%): 336/334 (94/100) [M^+], 185/183 (39/38) [$C_8H_8Br^+$], 135 (34) [$C_7H_5NS^+$], 104 (73) [$C_8H_8^+$], 93 (75) [$C_6H_7N^+$], 77 (44) [$C_6H_5^+$]. – EI-HRMS ($C_{15}H_{15}BrN_2S$): calcd. 334.0139, found 334.0143. – $C_{15}H_{15}BrN_2S$ (334): C 53.74, H 4.51, N 8.36, S 9.56, found C 53.61, H 4.21, N 8.41, S 9.74.

(*R*)-1-(1-Cyclopropylethyl)-3-phenylthiourea ((*R*)-9d):

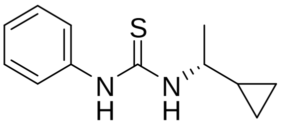

316 mg, 99% (scale: 1.45 mmol). – R_f (*c*-Hex/EtOAc 5:1) = 0.18. – mp: 54–59 °C. – $[\alpha]_D^{20}$ = −29.5 (*c* = 1.01, $CHCl_3$). – ^1H NMR (400 MHz, $CDCl_3$): δ/ppm = 0.26–0.31 (m, 1 H, CH_2), 0.40–0.45 (m, 2 H, CH_2), 0.47–0.53 (m, 1 H, CH_2), 0.77–0.86 (m, 1 H, C*H*), 1.24 (d, ^3J = 6.6 Hz, 3 H, CH_3), 3.94–4.03 (m, 1 H, NHC*H*), 6.03 (d, ^3J = 7.2 Hz, 1 H, N*H*), 7.21 (d, ^3J = 7.8 Hz, 2 H, 2 × H_{ar}), 7.26–7.31 (m, 1 H, H_{ar}), 7.41–7.45 (m, 2 H, 2 × H_{ar}), 7.96 (s, 1 H, Ar-N*H*). – ^{13}C NMR (100 MHz, $CDCl_3$): δ/ppm = 3.1 (s, 2 × CH_2), 16.9 (t, *C*H), 19.8 (p, CH_3), 55.5 (t, NH*C*H), 124.9 (t, C_{ar}H), 127.0 (t, 2 × C_{ar}H), 130.2 (t, 2 × C_{ar}H), 136.2 (q, C_{ar}), 179.3 (q, *C*=S). – IR (DRIFT): ṽ/cm^{-1} = 3379 (w), 3267 (w), 3079 (w), 3001 (w), 2928 (w), 1597 (w), 1524 (w), 1450 (w), 1351 (w), 1247 (w), 1176 (w), 1136 (w), 1021 (w), 1003 (w), 919 (w), 827 (w), 745 (w), 696 (w). – EI-MS *m/z* (%): 220 (37) [M^+], 191 (100) [$C_{10}H_{11}N_2S^+$], 177 (27), 93 (18) [$C_6H_7N^+$], 77 (11) [$C_6H_5^+$]. – EI-HRMS ($C_{12}H_{16}N_2S$): calcd. 220.1034, found

220.1032. – $C_{12}H_{16}N_2S$ (220.1): calcd. C 65.41, H 7.32, N 12.71, S 14.55, found C 65.23, H 6.92, N 12.83, S 14.31.

(*S*)-1-(1-(3-Chlorophenyl)ethyl)-3-phenylthiourea ((*S*)-9e):

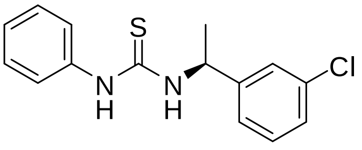

417 mg, 99% (scale: 1.45 mmol). – R_f (*c*-Hex/EtOAc 5:1) = 0.22. – $[\alpha]_D^{20}$ = 101 (*c* = 0.98, $CHCl_3$). – ^{1}H NMR (400 MHz, $CDCl_3$): δ/ppm = 1.50 (d, ^3J = 6.9 Hz, 3 H, CH_3), 5.66–5.68 (m, 1 H, C*H*), 6.19 (d, ^3J = 5.8 Hz, 1 H, N*H*), 7.17–7.22 (m, 3 H, 3 × H_{ar}), 7.23–7.26 (m, 3 H, 3 × H_{ar}), 7.28–7.32 (m, 1 H, H_{ar}), 7.41–7.45 (m, 2 H, 2 × H_{ar}), 8.08 (s, 1 H, Ar-N*H*). – ^{13}C NMR (100 MHz, $CDCl_3$): δ/ppm = 21.4 (p, CH_3), 53.8 (t, *C*H), 124.4 (t, C_{ar}H), 125.2 (t, 2 × C_{ar}H), 126.3 (t, C_{ar}H), 127.4 (t, C_{ar}H), 127.6 (t, C_{ar}H), 130.0 (t, C_{ar}H), 130.3 (t, 2 × C_{ar}H), 134.5 (q, C_{ar}-Cl), 135.9 (q, C_{ar}), 144.4 (q, C_{ar}), 179.9 (q, *C*=S). – IR (KBr): ṽ/cm^{-1} = 3376 (m), 3263 (m), 3256 (m), 2977 (m), 2929 (m), 1781 (m), 1596 (m), 1540 (m), 1450 (m), 1315 (m), 1259 (m), 1081 (w), 1028 (w), 1000 (w), 954 (w), 880 (w), 837 (w), 785 (w), 746 (w), 695 (w), 605 (w), 445 (w). – EI-MS *m/z* (%): 292/290 (37/100) [M^+], 156/154 (8/27) [$C_8H_9ClN^+$], 141/139 (11/32) [$C_8H_8Cl^+$], 93 (44) [$C_6H_7N^+$]. – EI-HRMS ($C_{15}H_{15}ClN_2S$): calcd. 290.0644, found 290.0642.

(*S*)-1-(1-(3-Bromophenyl)ethyl)-3-phenylthiourea ((*S*)-9f):

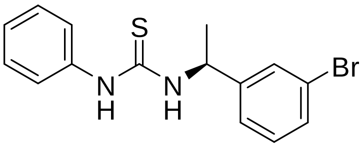

476 mg, 98% (scale: 1.45 mmol). – R_f (*c*-Hex/EtOAc 5:1) = 0.16. – $[\alpha]_D^{20}$ = 77.1 (*c* = 1.10, $CHCl_3$). – ^{1}H NMR (400 MHz, $CDCl_3$): δ/ppm = 1.49 (d, ^3J = 6.9 Hz, 3 H, CH_3), 5.65–5.67 (m, 1 H, C*H*), 6.18 (d, ^3J = 5.8 Hz, 1 H, N*H*), 7.17–7.25 (m, 4 H, 4 × H_{ar}), 7.29–7.34 (m, 1 H, H_{ar}), 7.36–7.45 (m, 4 H, 4 × H_{ar}), 8.08 (s, 1 H, Ar-N*H*). – ^{13}C NMR (100 MHz, $CDCl_3$): δ/ppm = 21.4 (p, CH_3), 53.8 (t, *C*H), 122.7 (q, C_{ar}-Br), 124.9 (t, C_{ar}H), 125.2 (t, 2 × C_{ar}H), 127.4 (t, C_{ar}H), 129.2 (t, 2 × C_{ar}H), 130.3 (t, C_{ar}H), 130.5 (t, 2 × C_{ar}H), 135.9 (q, C_{ar}), 144.7 (q, C_{ar}), 179.9 (q, *C*=S). – IR (KBr): ṽ/cm^{-1} = 3374 (m), 3241 (m), 3040 (m), 2976 (m), 1594 (m), 1569 (m), 1530 (s), 1496 (s), 1476 (m), 1499 (m), 1427 (m), 1375 (m), 1314 (s), 1244 (m), 1200 (m), 1137 (w), 1073 (m), 1028 (w), 997 (w), 953 (w), 909 (w), 880 (w), 823 (w), 783 (m), 695 (s), 672 (m), 605 (w), 502 (w), 438 (w). – EI-MS *m/z* (%): 336/334 (19/18) [M^+], 186/184 (40/49) [$C_7H_7BrN^+$], 185/183 (36/33) [$C_8H_8Br^+$], 118 (41), 105/103 (32/44) [C_2HBr^+],

93 (35) [$C_6H_7N^+$], 86/84 (57/87), 78/76 (43/23) [Br^+], 77 (98) [$C_6H_5^+$], 65 (32) [$C_5H_5^+$], 49 (36). – EI-HRMS ($C_{15}H_{15}BrN_2S$): calcd. 334.0139, found 334.0136.

(*R*)-1-(3,5-Bis(trifluoromethyl)phenyl)-3-(1-cyclohexylethyl)thiourea ((*R*)-9g) [1]:

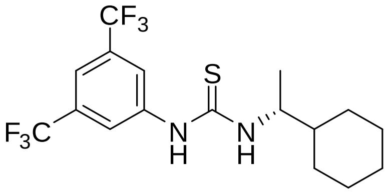

100 mg, 69% (scale: 361 µmol). – R_f (*c*-Hex/EtOAc 5:1) = 0.26. – mp: 166–170 °C. – $[\alpha]_D^{20}$ = −35.9 (*c* = 1.11, $CHCl_3$). – ^{1}H NMR (400 MHz, $CDCl_3$): δ/ppm = 0.91–1.29 (m, 5 H, C*H*$_2$), 1.17 (d, ^3J = 6.6 Hz, 3 H, C*H*$_3$), 1.43–1.51 (m, 1 H, C*H*), 1.64–1.70 (m, 2 H, C*H*$_2$), 1.75–1.78 (m, 3 H, C*H*$_2$), 4.37–4.39 (m, 1 H, NHC*H*), 6.02–6.03 (m, 1 H, N*H*), 7.71 (s, 1 H, H_{ar}), 7.74 (s, 2 H, 2 × H_{ar}), 8.53 (s, 1 H, Ar-N*H*). – ^{13}C NMR (100 MHz, $CDCl_3$): δ/ppm = 17.1 (p, *C*H$_3$), 25.9 (s, *C*H$_2$), 26.0 (s, *C*H$_2$), 26.2 (s, *C*H$_2$), 29.1 (s, *C*H$_2$), 29.2 (s, *C*H$_2$), 43.0 (t, *C*H), 55.9 (t, NH*C*H), 119.4 (t, sept, ^3J = 3.7 Hz, C_{ar}-4), 122.7 (q, quart, ^1J = 273.2 Hz, 2 × *C*F$_3$), 123.8 (t, quart, ^3J = 3.4 Hz, C_{ar}-2, C_{ar}-6), 133.2 (q, quart, ^2J = 36.3 Hz, C_{ar}-3, C_{ar}-5), 138.7 (q, C_{ar}-1), 179.6 (q, *C*=S). – ^{19}F NMR (376 MHz, $CDCl_3$): δ/ppm = −63.1 (s, 2 × C*F*$_3$). – IR (DRIFT): $\tilde{\nu}$/cm^{-1} = 3227 (m), 3062 (m), 2935 (s), 2856 (m), 1797 (w), 1623 (m), 1552 (s), 1468 (m), 1381 (s), 1348 (s), 1279 (s), 1187 (s), 1133 (s), 1107 (m), 1067 (w), 1044 (w), 1002 (m), 977 (m), 935 (m), 891 (s), 848 (m), 748 (m), 714 (m), 682 (m), 586 (m). – EI-MS *m/z* (%): 398 (62) [M^+], 289 (100) [$C_9H_7F_6N_2S^+$]. – EI-HRMS ($C_{17}H_{20}F_6N_2S$): calcd. 398.1251, found 398.1253.

(*S*)-1-(3,5-Bis-(trifluoromethyl)phenyl)-3-(1-cyclohexylethyl)thiourea ((*S*)-9g) [1]:

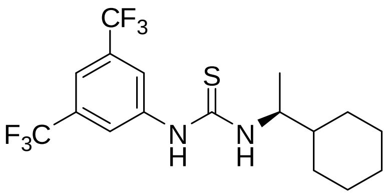

89.5 mg, 62% (scale: 361 µmol). – R_f (*c*-Hex/EtOAc 5:1) = 0.19. – mp: 169–173 °C. – $[\alpha]_D^{20}$ = 31.2 (*c* = 1.05, $CHCl_3$). – ^{1}H NMR (400 MHz, $CDCl_3$): δ/ppm = 0.91–1.29 (m, 5 H, C*H*$_2$), 1.18 (d, ^3J = 6.6 Hz, 3 H, C*H*$_3$), 1.43–1.52 (m, 1 H, C*H*), 1.60–1.69 (m, 2 H, C*H*$_2$), 1.72–1.83 (m, 3 H, C*H*$_2$), 4.16–4.67 (m, 1 H, NHC*H*), 5.84–6.24 (m, 1 H, N*H*CH), 7.71–7.74 (m, 3 H, 3 × H_{ar}), 8.39 (s, 1 H, Ar-N*H*). – ^{13}C NMR (100 MHz, $CDCl_3$): δ/ppm = 17.1 (p, *C*H$_3$), 25.9 (s, *C*H$_2$), 26.0 (s, *C*H$_2$), 26.2 (s, *C*H$_2$), 29.1 (s, *C*H$_2$), 29.2 (s, *C*H$_2$), 43.0 (t, *C*H), 56.0 (t, NH*C*H), 119.4 (t, sept, ^3J = 3.8 Hz, C_{ar}-4), 122.7 (q, quart, ^1J = 273.2 Hz, 2 × *C*F$_3$), 123.8 (t, C_{ar}-2, C_{ar}-6), 133.2 (t, quart, ^2J = 31.6 Hz, C_{ar}-3, C_{ar}-5), 138.7 (q, C_{ar}-1), 179.7 (q, *C*=S). – ^{19}F NMR (376 MHz, $CDCl_3$): δ/ppm = −63.1 (s, 2 × C*F*$_3$). –

IR (DRIFT): ṽ/cm^{-1} = 3226 (m), 3060 (m), 2934 (m), 2855 (m), 1622 (w), 1552 (m), 1430 (m), 1386 (m), 1348 (m), 1280 (s), 1187 (m), 1133 (s), 1107 (m), 1102 (m), 977 (m), 935 (w), 891 (m), 848 (w), 748 (w), 713 (m), 682 (m), 586 (w). – EI-MS *m/z* (%): 398 (19) [M^+], 289 (65) [$C_9H_7F_6N_2S^+$], 55 (22), 43 (100) [$C_2H_5N^+$]. – EI-HRMS ($C_{17}H_{20}F_6N_2S$): calcd. 398.1251, found 398.1253.

(*R*)-1-(3,5-Bis(trifluoromethyl)phenyl)-3-(1-phenylethyl)thiourea ((*R*)-9h):

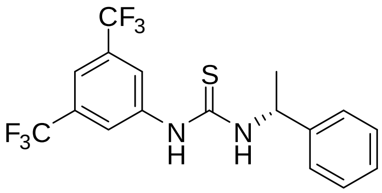

126 mg, 89% (scale: 361 µmol). – R_f (*c*-Hex/EtOAc 5:1) = 0.23. – mp: 139–143 °C. – $[\alpha]_D^{20}$ = 3.50 (*c* = 1.00, $CHCl_3$) – ^{1}H NMR (400 MHz, $CDCl_3$): δ/ppm = 1.60 (d, ^3J = 6.8 Hz, 3 H, C*H*$_3$), 5.36–5.44 (m, 1 H, C*H*), 6.57–6.65 (m, 1 H, N*H*), 7.34–7.42 (m, 5 H, 5 × H_{ar}), 7.65–7.68 (m, 3 H, 3 × H_{ar}), 8.08 (s, 1 H, Ar-N*H*). – ^{13}C NMR (100 MHz, $CDCl_3$): δ/ppm = 22.1 (p, *C*H$_3$), 54.8 (t, *C*H), 119.4 (t, sept, ^3J = 3.7 Hz, C_{ar}-4), 122.8 (q, quart, ^1J = 272.9 Hz, 2 × *C*F$_3$), 123.9 (t, quart, ^3J = 3.4 Hz, C_{ar}-2, C_{ar}-6), 126.1 (t, 2 × C_{ar}H), 128.4 (t, C_{ar}H), 129.3 (t, 2 × C_{ar}H), 132.6 (q, quart, ^2J = 34.3 Hz, C_{ar}-3, C_{ar}-5), 139.0 (q, C_{ar}-1), 141.3 (q, C_{ar}), 179.9 (q, *C*=S). – ^{19}F NMR (376 MHz, $CDCl_3$): δ/ppm = −63.1 (s, 2 × C*F*$_3$). – IR (DRIFT): ṽ/cm^{-1} = 3229 (m), 3042 (m), 2808 (w), 2656 (w), 2555 (w), 2234 (w), 2041 (m), 1992 (m), 1883 (w), 1806 (w), 1624 (m), 1544 (s), 1495 (m), 1469 (m), 1379 (m), 1341 (m), 1280 (s), 1227 (m), 1176 (m), 1134 (s), 1109 (m), 1063 (m), 1023 (m), 1002 (m), 976 (m), 893 (m), 848 (m), 791 (m), 767 (m), 743 (m), 704 (m), 682 (m), 620 (m), 587 (m), 232 (m), 456 (m), 405 (w). – EI-MS *m/z* (%): 392 (74) [M^+], 229 (18) [$C_8H_5F_6N^+$], 120 (25) [$C_8H_{10}N^+$], 77 (18) [$C_6H_5^+$]. – EI-HRMS ($C_{17}H_{14}F_6N_2S$): calcd. 392.0782, found 392.0780. – $C_{17}H_{14}F_6N_2S$ (392.1): calcd. C 52.04, H 3.60, N 7.14, S 8.17, found C 51.23, H 3.39, N 7.06, S 8.46.

(*S*)-1-(3,5-Bis(trifluoromethyl)phenyl)-3-(1-phenylethyl)thiourea ((*S*)-9h) [4]:

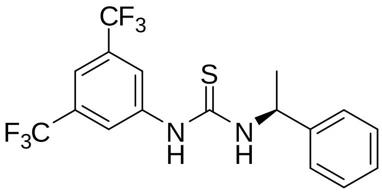

140 mg, 99% (scale: 361 µmol). – R_f (*c*-Hex/EtOAc 5:1) = 0.28. – mp: 137–140 °C. – $[\alpha]_D^{20}$ = −2.7 (*c* = 1.01, $CHCl_3$). – ^{1}H NMR (400 MHz, $CDCl_3$): δ/ppm = 1.60 (d, ^3J = 6.8 Hz, 3 H, C*H*$_3$), 4.77–5.94 (m, 1 H, C*H*), 6.14–7.00 (m, 1 H, N*H*), 7.32–7.42 (m, 5 H, 5 × H_{ar}), 7.63–7.68 (m, 3 H, 3 × H_{ar}), 7.99 (s, 1 H, Ar-N*H*). – ^{13}C NMR (100 MHz, $CDCl_3$): δ/ppm = 22.2 (p, *C*H$_3$), 54. 8 (t, *C*H), 119.4 (t, sept, ^3J = 3.6 Hz, C_{ar}-4), 122.7 (q, quart, ^1J = 273.2 Hz,

2 × CF_3), 123.9 (t, C_{ar}-2, C_{ar}-6), 126.0 (t, 2 × C_{ar}H), 128.3 (t, C_{ar}H), 129.3 (t, 2 × C_{ar}H), 132.7 (q, quart, ^2J = 33.7 Hz, C_{ar}-3, C_{ar}-5), 139.0 (q, C_{ar}-1), 141.2 (q, C_{ar}), 179.8 (q, *C*=S). – ^{19}F NMR (376 MHz, $CDCl_3$): δ/ppm = −63.1 (s, 2 × C*F*$_3$). – IR (DRIFT): ṽ/cm^{-1} = 3230 (m), 3042 (m), 2047 (w), 1805 (w), 1625 (m), 1544 (s), 1469 (m), 1381 (s), 1341 (m), 1283 (s), 1177 (s), 1135 (s), 1023 (m), 1002 (w), 976 (m), 893 (m), 848 (m), 791 (w), 767 (m), 743 (m), 704 (m), 682 (m), 532 (m). – EI-MS *m/z* (%): 392 (59) [M^+], 271 (44) [$C_9H_4F_6NS^+$], 213 (22) [$C_8H_3F_6^+$], 120 (23) [$C_8H_{10}N^+$], 105 (100) [$C_8H_9^+$], 77 (27) [$C_6H_5^+$]. – EI-HRMS ($C_{17}H_{14}F_6N_2S$): calcd. 392.0781, found 392.0784.

(*R*)-1-(3,5-Bis(trifluoromethyl)phenyl)-3-(1-(4-bromophenyl)ethyl)thiourea ((*R*)-9i):

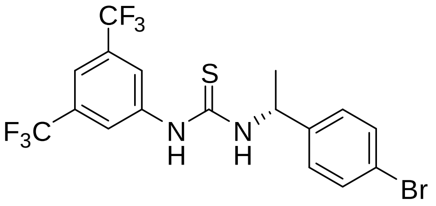

156 mg, 91% (scale: 361 µmol). – R_f (*c*-Hex/EtOAc 5:1) = 0.16. – mp: 127–131 °C. – $[\alpha]_D^{20}$ = −2.2 (*c* = 1.01, $CHCl_3$). – ^{1}H NMR (400 MHz, $CDCl_3$): δ/ppm = 1.56 (d, ^3J = 6.9 Hz, 3 H, C*H*$_3$), 5.41–5.52 (m, 1 H, C*H*), 6.35–6.51 (m, 1 H, N*H*), 7.21–7.23 (m, 2 H, 2 × H_{ar}), 7.49–7.51 (m, 2 H, 2 × H_{ar}), 7.65–7.72 (m, 3 H, 3 × H_{ar}), 8.07 (s, 1 H, Ar-N*H*). – ^{13}C NMR (100 MHz, $CDCl_3$): δ/ppm = 21.7 (p, *C*H$_3$), 54.1 (t, *C*H), 119.6 (t, sept, ^3J = 3.6 Hz, C_{ar}-4), 122.0 (q, C_{ar}-Br), 122.0 (t, C_{ar}-2, C_{ar}-6), 122.7 (q, quart, ^1J = 273.0 Hz, 2 × CF_3), 127.8 (t, 2 × C_{ar}H), 132.2 (t, 2 × C_{ar}H), 133.0 (q, quart, ^2J = 33.7 Hz, C_{ar}-3, C_{ar}-5), 138.7 (q, C_{ar}-1), 140.5 (q, C_{ar}), 179.9 (q, *C*=S). – ^{19}F NMR (376 MHz, $CDCl_3$): δ/ppm = −63.0 (s, 2 × C*F*$_3$). – IR (DRIFT): ṽ/cm^{-1} = 3232 (m), 3032 (s), 1799 (w), 1623 (m), 1541 (s), 1491 (s), 1468 (m), 1410 (m), 1380 (s), 1338 (s), 1278 (s), 1224 (m), 1174 (s), 1137 (s), 1108 (m), 1076 (m), 1032 (m), 1011 (m), 977 (m), 893 (s), 847 (m), 827 (m), 784 (m), 750 (m), 704 (s), 682 (s), 619 (w), 587 (w), 531 (m). – EI-MS *m/z* (%): 472/470 (100/97) [M^+], 271 (54) [$C_9H_4F_6NS^+$], 229 (26) [$C_8H_5F_6N^+$], 200/198 (20/19) [$C_8H_9BrN^+$], 185/183 (69/68) [$C_8H_8Br^+$], 104 (38) [$CH_4N_2S^+$]. – EI-HRMS ($C_{17}H_{13}BrF_6N_2S$): calcd. 469.9887, found 469.9886. – $C_{17}H_{13}BrF_6N_2S$ (470.0): calcd. C 43.33, H 2.78, N 5.94, S 6.80, found C 43.34, H 2.61, N 5.97, S 7.23.

(*S*)-1-(3,5-Bis(trifluoromethyl)phenyl)-3-(1-(4-bromophenyl)ethyl)thiourea ((*S*)-9i):

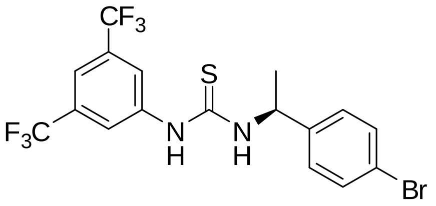

169 mg, 99% (scale: 361 µmol). – R_f (*c*-Hex/EtOAc 5:1) = 0.28. – mp: 130–133 °C. – $[\alpha]_D^{20}$ = −18.7 (*c* = 1.02, $CHCl_3$). – ^1H NMR (400 MHz, $CDCl_3$): δ/ppm = 1.56 (d, ^3J = 6.9 Hz, 3 H, CH_3), 5.36–5.53 (m, 1 H, C*H*), 6.32–6.55 (m, 1 H, N*H*), 7.21–7.23 (m, 2 H, 2 × H_{ar}), 7.49–7.51 (m, 2 H, 2 × H_{ar}), 7.65–7.70 (m, 3 H, 3 × H_{ar}), 8.04 (s, 1 H, Ar-N*H*). – ^{13}C NMR (100 MHz, $CDCl_3$): δ/ppm = 21.7 (p, CH_3), 54.1 (t, *C*H), 119.6 (t, sept, ^3J = 3.6 Hz, C_{ar}-4), 122.0 (q, C_{ar}-Br), 124.0 (t, C_{ar}-2, C_{ar}-6), 122.6 (q, quart, ^1J = 273.1 Hz, 2 × CF_3), 127.8 (t, 2 × C_{ar}H), 132.2 (t, 2 × C_{ar}H), 133.0 (q, quart, ^2J = 34.8 Hz, C_{ar}-3, C_{ar}-5), 138.7 (q, C_{ar}-1), 140.5 (q, C_{ar}), 179.9 (q, *C*=S). – ^{19}F NMR (376 MHz, $CDCl_3$): δ/ppm = −63.0 (s, 2 × CF_3). – IR (DRIFT): ṽ/cm^{-1} = 3238 (m), 3032 (m), 1623 (w), 1541 (s), 1491 (m), 1469 (m), 1680 (m), 1338 (m), 1279 (s), 1175 (m), 1137 (s), 1108 (m), 1076 (m), 1011 (m), 977 (m), 893 (m), 827 (m), 784 (w), 751 (w), 705 (m), 682 (m), 587 (w), 531 (m). – EI-MS *m/z* (%): 472/470 (23/23) [M^+], 271 (31) [$C_9H_4F_6NS^+$], 213 (35) [$C_8H_3F_6^+$], 185/183 (45/49) [$C_8H_9Br^+$], 184/182 (35/31) [$C_8H_8Br^+$], 104/102 (100/15) [C_2HBr^+], 78/76 (23/11) [Br^+]. – EI-HRMS ($C_{17}H_{13}BrF_6N_2S$): calcd. 469.9887, found 469.9884.

(*R*)-1-(3,5-Bis(trifluoromethyl)phenyl)-3-(1-cyclopropylethyl)thiourea ((*R*)-9j):

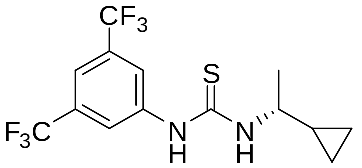

117 mg, 91% (scale: 361 µmol). – R_f (*c*-Hex/EtOAc 5:1) = 0.20. – mp: 160–164 °C. – $[\alpha]_D^{20}$ = −38.5 (*c* = 1.01, $CHCl_3$). – ^1H NMR (400 MHz, $CDCl_3$): δ/ppm = 0.30–0.36 (m, 1 H, CH_2), 0.41–0.47 (m, 1 H, CH_2), 0.50–0.62 (m, 2 H, CH_2), 0.85–0.94 (m, 1 H, C*H*), 1.30 (d, ^3J = 6.6 Hz, 3 H, CH_3), 3.77–4.02 (m, 1 H, NHC*H*), 6.09–6.32 (m, 1 H, N*H*), 7.68–7.73 (m, 1 H, H_{ar}), 7.74–7.77 (m, 2 H, 2 × H_{ar}), 8.44 (s, 1 H, Ar-N*H*). – ^{13}C NMR (100 MHz, $CDCl_3$): δ/ppm = 3.2 (s, CH_2), 3.3 (s, CH_2), 16.8 (t, *C*H), 19.5 (p, CH_3), 55.8 (t, NH*C*H), 119.2 (t, sept, ^3J = 3.7 Hz, C_{ar}-4), 122.7 (q, quart, ^1J = 273.0 Hz, 2 × CF_3), 132.6 (t, C_{ar}-2, C_{ar}-6), 133.2 (q, quart, ^2J = 33.7 Hz, C_{ar}-3, C_{ar}-5), 138.9 (q, C_{ar}-1), 179.4 (q, *C*=S). – ^{19}F NMR (376 MHz, $CDCl_3$): δ/ppm = −63.1 (s, 2 × CF_3). – IR (DRIFT): ṽ/cm^{-1} = 3242 (m), 3048 (m), 1797 (w), 1624 (m), 1553 (s), 1468 (m), 1382 (m), 1348 (m), 1280 (s), 1227 (m), 1192 (s), 1133 (s), 1108 (m), 1053 (m), 1026 (m), 1013 (m), 976 (m), 939 (m), 890 (m), 848 (m), 834 (w), 748 (w), 715 (m), 704 (m), 682 (m), 620 (w), 591 (w).

– EI-MS *m/z* (%): 356 (28) [M^+], 327 (100) [$C_{12}H_9F_6N_2S^+$], 313 (39) [$C_{11}H_7F_6N_2S^+$], 41 (18) [$C_3H_5^+$]. – EI-HRMS ($C_{14}H_{14}F_6N_2S$): calcd. 356.0779, found 356.0782.

(*S*)-1-(3,5-Bis(trifluoromethyl)phenyl)-3-(1-(3-chlorophenyl)ethyl)thiourea ((*S*)-9k):

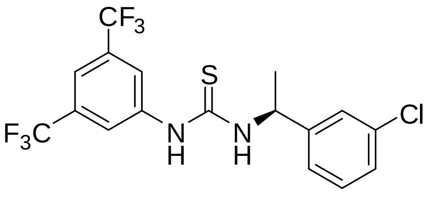

153 mg, 99% (scale: 361 µmol). – R_f (*c*-Hex/EtOAc 5:1) = 0.20. – mp: 129–131 °C. – $[\alpha]_D^{20}$ = 48.7 (*c* = 1.01, $CHCl_3$). – ^{1}H NMR (400 MHz, $CDCl_3$): δ/ppm = 1.57 (d, ^3J = 6.9 Hz, 3 H, C*H*$_3$), 5.32–5.62 (m, 1 H, C*H*), 6.21–6.59 (m, 1 H, N*H*), 7.22–7.24 (m, 1 H, H_{ar}), 7.27–7.33 (m, 3 H, 3 × H_{ar}), 7.69–7.71 (m, 3 H, 3 × H_{ar}), 8.03 (s, 1 H, Ar-N*H*). – ^{13}C NMR (100 MHz, $CDCl_3$): δ/ppm = 21.7 (p, *C*H$_3$), 54.2 (t, *C*H), 119.7 (t, sept, ^3J = 3.4 Hz, C_{ar}-4), 122.7 (q, quart, ^1J = 273.0 Hz, 2 × *C*F$_3$), 124.0 (t, C_{ar}-2, C_{ar}-6), 124.4 (t, C_{ar}H), 126.3 (t, C_{ar}H), 128.3 (t, C_{ar}H), 130.4 (t, C_{ar}H), 133.0 (q, quart, ^2J = 34.8 Hz, C_{ar}-3, C_{ar}-5), 135.0 (q, C_{ar}-Cl), 138.9 (q, C_{ar}-1), 143.5 (q, C_{ar}), 179.9 (q, *C*=S). – ^{19}F NMR (376 MHz, $CDCl_3$): δ/ppm = −63.1 (s, 2 × C*F*$_3$). – IR (DRIFT): ṽ/cm^{-1} = 3226 (m), 3054 (m), 1807 (w), 1623 (w), 1549 (m), 1471 (m), 1431 (m), 1381 (m), 1341 (m), 1278 (m), 1231 (m), 1186 (m), 1130 (m), 1080 (m), 1032 (w), 1000 (w), 977 (m), 914 (w), 891 (m), 866 (w), 848 (w), 785 (m), 752 (w), 712 (m), 683 (m), 594 (w), 459 (w), 444 (w). – EI-MS *m/z* (%): 428/426 (17/43) [M^+], 287 (30) [$C_9H_5F_6N_2S^+$], 272 (22) [$C_9H_4F_6NS^+$], 229 (41) [$C_8H_5F_6N^+$], 228 (25) [$C_8H_4F_6N^+$], 213 (37) [$C_8H_3F^+$], 156/154 (14/44) [$C_8H_9ClN^+$], 141/139 (27/97) [$C_8H_8Cl^+$], 140/138 (19/29) [$C_8H_7Cl^+$], 103 (100) [$C_3H_7N_2S^+$], 77 (53) [$C_6H_5^+$], 43 (98) [$C_2H_5N^+$]. – EI-HRMS ($C_{17}H_{13}ClF_6N_2S$): calcd. 426.0392, found 426.0395.

(*S*)-1-(3,5-Bis(trifluoromethyl)phenyl)-3-(1-(3-bromophenyl)ethyl)thiourea ((*S*)-9l):

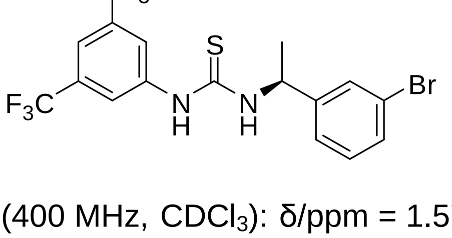

130 mg, 76% (scale: 361 µmol). – R_f (*c*-Hex/EtOAc 5:1) = 0.20. – mp: 143–149 °C. – $[\alpha]_D^{20}$ = 36.5 (*c* = 0.97, $CHCl_3$). – ^{1}H NMR (400 MHz, $CDCl_3$): δ/ppm = 1.57 (d, ^3J = 6.9 Hz, 3 H, C*H*$_3$), 5.34–5.60 (m, 1 H, C*H*), 6.30–6.56 (m, 1 H, N*H*), 7.23–7.29 (m, 2 H, 2 × H_{ar}), 7.43–7.47 (m, 2 H, 2 × H_{ar}), 7.69–7.74 (m, 3 H, 3 × H_{ar}), 8.03 (s, 1 H, Ar-N*H*). – ^{13}C NMR (100 MHz, $CDCl_3$): δ/ppm = 21.7 (p, *C*H$_3$), 54.1 (t, *C*H), 119.7 (t, sept, ^3J = 3.7 Hz, C_{ar}-4), 122.6 (q, quart,

^{1}J = 273.1 Hz, 2 × CF_3), 123.2 (q, C_{ar}-Br), 124.0 (t, C_{ar}-2), 124.9 (t, C_{ar}-6), 129.2 (t, C_{ar}H), 130.7 (t, 2 × C_{ar}H), 131.3 (t, 2 × C_{ar}H), 133.0 (q, quart, ^{2}J = 34.8 Hz, C_{ar}-3, C_{ar}-5), 138.7 (q, C_{ar}-1), 143.8 (q, C_{ar}), 179.9 (q, *C*=S). – ^{19}F NMR (376 MHz, $CDCl_3$): δ/ppm = −63.0 (s, 2 × CF_3). – IR (DRIFT): ṽ/cm^{-1} = 3326 (m), 3053 (m), 1947 (w), 1807 (w), 1623 (m), 1549 (s), 1471 (m), 1425 (m), 1381 (m), 1341 (m), 1294 (m), 1277 (m), 1232 (m), 1185 (s), 1129 (s), 1078 (m), 1030 (m), 998 (m), 976 (m), 914 (m), 890 (m), 867 (w), 849 (m), 822 (w), 784 (m), 752 (m). 708 (m), 682 (m), 593 (w), 458 (w), 437 (w). – EI-MS *m/z* (%): 473/471 (47/45) [M^+], 472/470 (100/99) [M^+−H], 316 (26) [$C_{11}H_{10}F_6N_2S^+$], 315 (22) [$C_{11}H_9F_6N_2S^+$], 287 (72) [$C_9H_5F_6N_2S^+$], 271 (52) [$C_9H_4F_6NS^+$]. – EI-HRMS ($C_{17}H_{13}BrF_6N_2S$): calcd. 469.9887, found 469.9890.

(*S*)-Phenylalanin-*N*-methylamide (13):

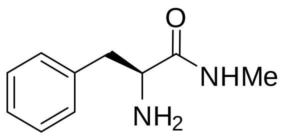

To a solution of methylamine (13.5 mL, 33% in EtOH, 45.9 mmol, 4.00 equiv) (*S*)-phenylalanine methyl ester hydrochloride (**12**, 2.50 g, 11.5 mmol, 1.00 equiv) was added, and the mixture was stirred for 2 d at room temperature. After removal of the solvent saturated $NaHCO_3$ (20 mL) was added and the free amine was extracted with dichloromethane (5 × 15 mL) and dried over Na_2SO_4, and the solvent evaporated.

2.02 g, 99%. R_f = 0.14 (EtOAc). – mp: 52–57 °C. – $[\alpha]_D^{20}$ = −48.14 (*c* = 1.08, $CHCl_3$). – ^{1}H NMR (400 MHz, methanol-d_4): δ/ppm = 2.66 (s, 3 H, CH_3), 2.79 (dd, ^{2}J = 13.4 Hz, ^{3}J = 7.2 Hz, 1 H, CH_2), 2.97 (dd, ^{2}J = 13.4 Hz, ^{3}J = 6.7 Hz, 1 H, CH_2), 3.49 (dd, ^{3}J = 7.2 Hz, ^{3}J = 6.7 Hz, 1 H, C*H*), 7.19–7.23 (m, 3 H, 2-H_{ar}, 4-H_{ar}, 6-H_{ar}), 7.27–7.30 (m, 2 H, 3-H_{ar}, 5-H_{ar}). – ^{13}C NMR (100 MHz, methanol-d_4): δ/ppm = 26.1 (p, CH_3), 42.6 (s, CH_2), 57.9 (t, *C*H), 127.8 (t, C_{ar}-2, C_{ar}-6), 129.6 (t, C_{ar}-4), 130.4 (t, C_{ar}-3, C_{ar}-5), 139.0 (q, C_{ar}-1), 177.3 (q, *C*=O). – IR (DRIFT): ṽ/cm^{-1} = 3372 (m), 3293 (m), 3086 (w), 3063 (w), 3033 (w), 2940 (m), 2915 (m), 2877 (m), 2791 (w), 1959 (w), 1773 (m), 1646 (m), 1528 (m), 1455 (m), 1401 (m), 1343 (w), 1269 (w), 1230 (m), 1160 (m), 1109 (w), 1082 (w), 1067 (w), 1031 (w), 1003 (w), 978 (w), 927 (m), 877 (m), 858 (m), 747 (m), 702 (m), 660 (w), 567 (w), 534 (w), 483 (m). – EI-MS (70 eV) *m/z* (%): 178 (2) [M^+], 120 (100) [M^+−C_2H_4NO], 91 (12) [$C_7H_7^+$], 87 (22) [M^+−C_7H_7]. – EI-HRMS ($C_{10}H_{14}N_2O$): calcd. 178.1110, found 178.1106.

General procedure for the preparation of Imidazolidinone-catalysts 15

1.00 equiv of (*S*)-phenylalanin-*N*-methylamide (**13**), 1.00 equiv aldehyde **14**, 0.30 equiv $FeCl_3$, and 3 Å MS were solved/suspended in THF (2 mL/mmol aldehyde). The resulting mixture was heated under reflux for 24 h, filtered off and the solvent was removed under reduced pressure. The product was purified by flash column chromatography.

(2*R*,5*S*)-5-Benzyl-2-*tert*-butyl-3-methylimidazolidin-4-one ((2*R*,5*S*)-15a) and (2*S*,5*S*)-5-benzyl-2-*tert*-butyl-3-methylimidazolidin-4-one ((2*S*,5*S*)-15a) [5]:

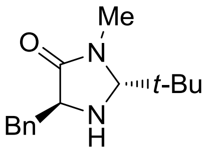

(2*R*,5*S*)-**15a**

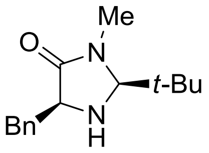

(2*S*,5*S*)-**15a**

Scale: 5.00 mmol. (2*R*,5*S*)-**15a:** 428 mg, 35%. – R_f (*c*-Hex/EtOAc 4:1) = 0.10. – mp: 92–94 °C. – $[\alpha]_D^{20}$ = −80.3 (*c* = 1.08, $CHCl_3$). – ^{1}H NMR (600 MHz, $CDCl_3$): δ/ppm = 0.90 (s, 9 H, $C(CH_3)_3$), 1.88 (s, 1 H, N*H*), 2.89 (s, 3 H, NCH_3), 2.89 (dd, ^2J = 14.1 Hz, ^3J = 4.2 Hz, 1 H, CH_2), 3.11 (dd, ^2J = 14.1 Hz, ^3J = 7.2 Hz, 1 H, CH_2), 3.80 (d, ^4J = 1.7 Hz, 1 H, NC*H*N), 3.85 (ddd, ^3J = 7.2 Hz, ^3J = 4.2 Hz, ^4J = 1.7 Hz, 1 H, CH_2CH), 7.22–7.25 (m, 3 H, 3 × H_{ar}), 7.28–7.31 (m, 2 H, 2 × H_{ar}). – ^{13}C NMR (100 MHz, $CDCl_3$): δ/ppm = 25.6 (p, 3 × $C(CH_3)_3$), 31.3 (p, NCH_3), 37.7 (q, $C(CH_3)_3$), 38.5 (s, CH_2), 59.5 (t, CH_2CH), 83.4 (t, NCHN), 126.6 (t, C_{ar}H), 128.5 (t, 2 × C_{ar}H), 129.5 (t, 2 × C_{ar}H), 137.4 (q, C_{ar}), 175.3 (q, *C*=O). – IR (DRIFT): ṽ/cm^{-1} = 3059 (w), 3030 (w), 1897 (w), 1682 (s), 1584 (w), 1457 (m), 1433 (m), 1397 (m), 1346 (m), 1330 (m), 1307 (m), 1283 (m), 1262 (m), 1204 (m), 1160 (m), 1104 (m), 1025 (w), 902 (m), 801 (w), 756 (m), 736 (m), 704 (m), 637 (w), 541 (m), 489 (m). – EI-MS *m/z* (%): 247 (3) [M^++H], 189 (100) [M^+−*t*-Bu]. – EI-HRMS ($C_{15}H_{22}N_2O+H^+$): calcd. 247.1810, found 247.1807. (2*S*,5*S*)-**15a:** 304 mg, 25%. – R_f (*c*-Hex/EtOAc 4:1) = 0.04. – mp: 82–84 °C. – $[\alpha]_D^{20}$ = −68.5 (*c* = 1.06, $CHCl_3$). – ^{1}H NMR (400 MHz, $CDCl_3$): δ/ppm = 0.83 (s, 9 H, $C(CH_3)_3$), 1.69 (s, 1 H, N*H*), 2.91 (s, 3 H, NCH_3), 2.89 (dd, ^2J = 13.8 Hz, ^3J = 7.2 Hz, 1 H, CH_2), 3.15 (dd, ^2J = 13.8 Hz, ^3J = 3.9 Hz, 1 H, CH_2), 3.68–3.73 (m, 1 H, CH_2CH), 4.05 (d, ^4J = 1.7 Hz, 1 H, NC*H*N), 7.21–7.31 (m, 5 H, 5 × H_{ar}). – ^{13}C NMR (100 MHz, $CDCl_3$): δ/ppm = 25.3 (p, 3 × $C(CH_3)_3$), 30.7 (p, NCH_3), 35.0 (q, $C(CH_3)_3$), 38.2 (s, CH_2), 59.4 (t, CH_2CH), 82.4 (t, NCHN), 126.6 (t, C_{ar}H), 128.5 (t, 2 × C_{ar}H), 129.6 (t, 2 × C_{ar}H), 137.8 (q, C_{ar}), 175.2 (q, *C*=O). – IR (DRIFT): ṽ/cm^{-1} = 3368 (m), 3355 (m), 3084 (w), 3060 (w), 3030 (w), 1893 (m), 1676 (s), 1603 (w), 1477 (m), 1421 (m), 1393 (m), 1360 (m), 1341 (m), 1261 (m), 1206 (m), 1107 (m), 1078 (w), 1051 (w),

1028 (w), 1008 (w), 974 (w), 946 (w), 913 (w), 892 (m), 827 (w), 792 (w), 764 (m), 740 (m), 710 (m), 700 (m), 617 (w), 574 (w), 543 (w), 489 (m), 421 (w), 412 (w). – EI-MS *m/z* (%): 247 (1) [M^++H], 189 (100) [M^+−*t*-Bu]. – EI-HRMS ($C_{15}H_{22}N_2O+H^+$): calcd. 247.1810, found 247.1813.

(2*R*,5*S*)-5-Benzyl-3-methyl-2-(2-nitrophenyl)imidazolidin-4-one ((2*R*,5*S*)-15b) and (2*S*,5*S*)-5-benzyl-3-methyl-2-(2-nitrophenyl)imidazolidin-4-one ((2*S*,5*S*)-15b):

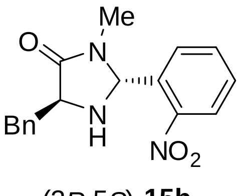

(2*R*,5*S*)-**15b**

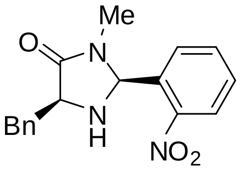

(2*S*,5*S*)-**15b**

Scale: 1.12 mmol. (2*R*,5*S*)-**15b:** 135 mg, 42%. – R_f (*c*-Hex/EtOAc 1:1) = 0.10. – $[\alpha]_D^{20}$ = 58.3 (*c* = 1.03, $CHCl_3$). – ^{1}H NMR (600 MHz, $CDCl_3$): δ/ppm = 2.56 (bs, 1 H, N*H*), 2.68 (s, 3 H, CH_3), 3.01 (dd, ^2J = 13.9 Hz, ^3J = 7.1 Hz, 1 H, CH_2), 3.15 (dd, ^2J = 13.9 Hz, ^3J = 4.1 Hz, 1 H, CH_2), 3.90–3.94 (m, 1 H, CH_2C*H*), 5.55 (d, ^4J = 1.3 Hz, 1 H, NC*H*N), 7.23–7.28 (m, 3 H, 3 × H_{ar}), 7.30–7.36 (m, 3 H, 3 × H_{ar}), 7.47–7.51 (m, 1 H, H_{ar}), 7.63–7.67 (m, 1 H, H_{ar}), 7.94–7.97 (m, 1 H, H_{ar}). – ^{13}C NMR (125 MHz, $CDCl_3$): δ/ppm = 27.7 (p, NCH_3), 38.3 (s, CH_2), 59.0 (t, CH_2*C*H), 72.1 (t, N*C*HN), 125.2 (t, C_{ar}H), 127.0 (t, C_{ar}H), 127.2 (t, C_{ar}H), 128.7 (t, 2 × C_{ar}H), 129.4 (t, 2 × C_{ar}H), 129.6 (t, C_{ar}H), 133.9 (t, C_{ar}H), 134.6 (q, C_{ar}), 136.8 (q, C_{ar}), 149.2 (q, C_{ar}-NO_2), 174.3 (q, *C*=O). – IR (KBr): ṽ/cm^{-1} = 3458 (m), 3374 (m), 3063 (w), 3029 (w), 2923 (w), 1690 (m), 1606 (w), 1581 (w), 1528 (m), 1496 (w), 1479 (w), 1453 (m), 1401 (m), 1349 (m), 1303 (w), 1262 (w), 1097 (w), 1079 (w), 1046 (w), 999 (w), 856 (w), 827 (w), 789 (w), 746 (w), 703 (w), 487 (w). – EI-MS *m/z* (%): 312 (92) [M^++H], 293 (33), 220 (100) [M^+−C_7H_7], 91 (27) [$C_7H_7^+$]. – EI-HRMS ($C_{17}H_{17}N_3O_3$): calcd. 312.1348, found 312.1346. (2*S*,5*S*)-**15b:** 155 mg, 52%. – R_f (*c*-Hex/EtOAc 1:1) = 0.25. – $[\alpha]_D^{20}$ = −165 (*c* = 1.04, $CHCl_3$). – ^{1}H NMR (600 MHz, $CDCl_3$): δ/ppm = 2.49 (bs, 1 H, N*H*), 2.62 (s, 3 H, CH_3), 3.07 (dd, ^2J = 13.8 Hz, ^3J = 6.3 Hz, 1 H, CH_2), 3.11 (dd, ^2J = 13.8 Hz, ^3J = 4.4 Hz, 1 H, CH_2), 4.00–4.02 (m, 1 H, CH_2C*H*), 5.79 (d, ^4J = 1.2 Hz, 1 H, NC*H*N), 6.71–6.73 (m, 1 H, H_{ar}), 7.18–7.20 (m, 2 H, 2 × H_{ar}), 7.23–7.26 (m, 3 H, 3 × H_{ar}), 7.43–7.46 (m, 2 H, 2 × H_{ar}), 7.85–7.88 (m, 1 H, H_{ar}). – ^{13}C NMR (100 MHz, $CDCl_3$): δ/ppm = 27.7 (p, NCH_3), 38.3 (s, CH_2), 60.0 (t, CH_2*C*H), 71.4 (t, N*C*HN), 124.3 (t, C_{ar}H), 126.6 (t, C_{ar}H), 128.3 (t, C_{ar}H), 128.5 (t, 2 × C_{ar}H), 129.5 (t, C_{ar}H), 130.0 (t, 2 × C_{ar}H), 133.7 (t, C_{ar}H), 134.4 (q, C_{ar}), 137.3 (q, C_{ar}), 149.7 (q, C_{ar}-NO_2), 173.7 (q, *C*=O). – IR (KBr): ṽ/cm^{-1} = 3472 (w), 3348 (w),

3063 (w), 3030 (w), 2922 (w), 1709 (m), 1688 (w), 1678 (w), 1607 (w), 1529 (w). – EI-MS *m/z* (%): 312 (8) [M^++H], 220 (32) [$M^+-C_7H_7$], 117 (24), 91 (100) [$C_7H_7^+$]. – EI-HRMS ($C_{17}H_{17}N_3O_3$): calcd. 312.1348, found 312.1346.

(2*R*,5*S*)-5-Benzyl-2-(3-fluorophenyl)-3-methylimidazolidin-4-one ((2*R*,5*S*)-15c) and (2*S*,5*S*)-5-benzyl-2-(3-fluorophenyl)-3-methylimidazolidin-4-one ((2*S*,5*S*)-15c):

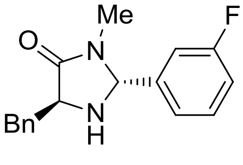

(2*R*,5*S*)-**15c**

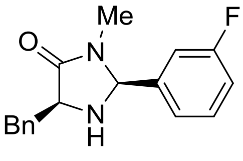

(2*S*,5*S*)-**15c**

Scale: 1.12 mmol. (2*R*,5*S*)-**15c:** 89.1 mg, 28%. – R_f (*c*-Hex/EtOAc 2:1) = 0.12. – $[\alpha]_D^{20}$ = −5.45 (*c* = 1.01, $CHCl_3$). – ^{1}H NMR (600 MHz, $CDCl_3$): δ/ppm = 2.15 (bs, 1 H, N*H*), 2.59 (s, 3 H, C*H*$_3$), 2.97 (dd, ^2J = 13.8 Hz, ^3J = 7.3 Hz, 1 H, C*H*$_2$), 3.12 (dd, ^2J = 13.8 Hz, ^3J = 4.0 Hz, 1 H, C*H*$_2$), 4.02–4.07 (m, 1 H, CH_2C*H*), 4.85 (d, ^4J = 1.0 Hz, 1 H, NC*H*N), 6.93–6.96 (m, 1 H, H_{ar}), 7.00–7.06 (m, 2 H, 2 × H_{ar}), 7.23–7.26 (m, 1 H, H_{ar}). 7.28–7.36 (m, 5 H, 5 × H_{ar}). – ^{13}C NMR (100 MHz, $CDCl_3$): δ/ppm = 27.2 (p, N*C*H_3), 38.6 (s, *C*H_2), 59.8 (t, CH_2*C*H), 77.2 (t, N*C*HN), 113.6 (t, dubl, ^2J = 21.9 Hz, $C_{ar'}$-4), 116.3 (t, dubl, ^2J = 21.2 Hz, $C_{ar'}$-2), 122.3 (t, $C_{ar'}$-6), 126.8 (t, C_{ar}H), 128.5 (t, 2 × C_{ar}H), 129.6 (t, 2 × C_{ar}H), 130.8 (t, dubl, ^3J = 8.1 Hz, $C_{ar'}$-5), 137.4 (q, C_{ar}), 142.0 (q, dubl, ^3J = 6.1 Hz, $C_{ar'}$-1), 163.1 (q, dubl, ^1J = 247.9 Hz, $C_{ar'}$-3), 174.1 (q, *C*=O). – ^{19}F NMR (376 MHz, $CDCl_3$): δ/ppm = −112.0 (s, *F*). – IR (KBr): ṽ/cm^{-1} = 3324 (s), 3084 (m), 3061 (s), 3029 (s), 2923 (s), 2866 (s), 1951 (w), 1883 (w), 1692 (s), 1614 (m), 1594 (m), 1453 (m), 1400 (m), 1359 (m), 1260 (m), 1139 (m), 1096 (m), 1030 (m), 1003 (m), 970 (m), 948 (m), 945 (m), 789 (m), 741 (m), 700 (w), 639 (w), 607 (w), 581 (w), 522 (w), 488 (w), 454 (w), 429 (w). – EI-MS *m/z* (%): 284 (9) [M^+], 193 (100) [$M^+-C_7H_7$], 138 (32) [$C_8H_9FN^+$]. – EI-HRMS ($C_{17}H_{17}FN_2O$): calcd. 284.1325, found 284.1323. (2*S*,5*S*)-**15c:** 124 mg, 39%. – R_f (*c*-Hex/EtOAc 2:1) = 0.04. – $[\alpha]_D^{20}$ = −91.2 (*c* = 1.00, $CHCl_3$). – ^{1}H NMR (600 MHz, $CDCl_3$): δ/ppm = 1.92 (bs, 1 H, N*H*), 2.55 (s, 3 H, C*H*$_3$), 3.11 (dd, ^2J = 14.0 Hz, ^3J = 4.6 Hz, 1 H, C*H*$_2$), 3.24 (dd, ^2J = 14.0 Hz, ^3J = 5.60 Hz, 1 H, C*H*$_2$), 3.86–3.89 (m, 1 H, CH_2C*H*), 5.12 (d, ^4J = 1.0 Hz, 1 H, NC*H*N), 6.43–6.46 (m, 1 H, H_{ar}), 6.66–6.69 (m, 1 H, H_{ar}), 6.98–7.03 (m, 1 H, H_{ar}). 7.20–7.24 (m, 3 H, 3 × H_{ar}), 7.28–7.33 (m, 3 H, 3 × H_{ar}). – ^{13}C NMR (100 MHz, $CDCl_3$): δ/ppm = 27.3 (p, N*C*H_3), 36.9 (s, *C*H_2), 60.2 (t, CH_2*C*H), 76.9 (t, dubl, ^4J = 1.5 Hz, N*C*HN), 113.8 (t, dubl, ^2J = 22.2 Hz, $C_{ar'}$-4), 116.5 (t, dubl, ^2J = 21.2 Hz, $C_{ar'}$-2), 122.9 (t, dubl, ^4J = 2.9 Hz, t, $C_{ar'}$-6), 127.1 (t,

C_{ar}H), 128.8 (t, 2 × C_{ar}H), 129.8 (t, 2 × C_{ar}H), 130.5 (t, dubl, ^3J = 8.1 Hz, $C_{ar'}$-5), 136.5 (q, C_{ar}), 141.2 (q, dubl, ^3J = 6.5 Hz, $C_{ar'}$-1), 163.0 (q, dubl, ^1J = 247.7 Hz, $C_{ar'}$-3), 174.2 (q, *C*=O). – ^{19}F NMR (400 MHz, $CDCl_3$): δ/ppm = −111.6 (s, *F*). – IR (KBr): ṽ/cm^{-1} = 3465 (m), 3339 (m), 3085 (m), 3062 (m), 3029 (m), 2923 (m), 2855 (m), 1689 (m), 1614 (m), 1595 (m), 1548 (w), 1484 (m), 1443 (m), 1402 (m), 1365 (m), 1335 (m), 1262 (m), 1180 (w), 1139 (m), 1098 (m), 1030 (w), 1003 (w), 955 (w), 913 (w), 882 (w), 788 (m), 746 (m), 701 (m), 597 (m), 521 (w), 484 (w), 454 (w), 430 (w). – EI-MS *m/z* (%): 284 (8) [M^+], 193 (100) [$M^+{-}C_7H_7$], 43 (52) [$C_2H_3O^+$]. – EI-HRMS ($C_{17}H_{17}FN_2O$): calcd. 284.1325, found 284.1323.

(2*R*,5*S*)-5-Benzyl-2-(4-fluorophenyl)-3-methylimidazolidin-4-one ((2*R*,5*S*)-15d) and (2*S*,5*S*)-5-benzyl-2-(4-fluorophenyl)-3-methylimidazolidin-4-one ((2*S*,5*S*)-15d):

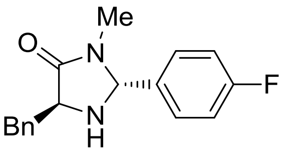

(2*R*,5*S*)-**15d**

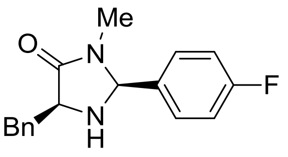

(2*S*,5*S*)-**15d**

Scale: 1.12 mmol. (2*R*,5*S*)-**15d:** 101 mg, 32%. – R_f (*c*-Hex/EtOAc 1:1) = 0.28. – $[\alpha]_D^{20}$ = −8.98 (*c* = 1.08, $CHCl_3$). – ^{1}H NMR (600 MHz, $CDCl_3$): δ/ppm = 2.14 (bs, 1 H, N*H*), 2.58 (s, 3 H, CH_3), 2.98 (dd, ^2J = 13.8 Hz, ^3J = 7.3 Hz, 1 H, CH_2), 3.14 (dd, ^2J = 13.8 Hz, ^3J = 4.0 Hz, 1 H, CH_2), 4.06–4.08 (m, 1 H, CH_2C*H*), 4.85 (d, ^4J = 1.7 Hz, 1 H, NC*H*N), 7.06–7.08 (m, 2 H, 2 × H_{ar}), 7.22–7.28 (m, 3 H, 3 × H_{ar}), 7.30–7.33 (m, 4 H, 4 × H_{ar}). – ^{13}C NMR (100 MHz, $CDCl_3$): δ/ppm = 27.1 (p, NCH_3), 38.7 (s, CH_2), 60.0 (t, CH_2*C*H), 77.0 (t, N*C*HN), 116.1 (t, dubl, ^2J = 21.9 Hz, $C_{ar'}$-3, $C_{ar'}$-5), 126.7 (t, C_{ar}H), 128.4 (t, 2 × C_{ar}H), 128.5 (t, dubl, ^3J = 8.6 Hz, $C_{ar'}$-2, $C_{ar'}$-6), 129.6 (t, 2 × C_{ar}H), 135.1 (q, dubl, ^4J = 3.1 Hz, $C_{ar'}$-1), 137.5 (q, C_{ar}), 163.1 (q, dubl, ^1J = 249 Hz, C_{ar}-F), 174.1 (q, *C*=O). – ^{19}F NMR (376 MHz, $CDCl_3$): δ/ppm = −112.0 (s, *F*). – IR (KBr): ṽ/cm^{-1} = 3474 (m), 3327 (m), 3062 (m), 3029 (m), 2922 (m), 1695 (s), 1605 (s), 1510 (s), 1479 (m), 1432 (s), 1400 (s), 1357 (m), 1263 (m), 1224 (s), 1157 (m), 1030 (w), 1029 (w), 1014 (w), 998 (w), 841 (m), 743 (w), 702 (w), 555 (w), 502 (w), 488 (w). – EI-MS *m/z* (%): 284 (9) [M^+], 193 (100) [$M^+{-}C_7H_7$], 58 (36) [$C_2H_4NO^+$], 43 (99) [$C_2H_3O^+$]. – EI-HRMS ($C_{17}H_{17}FN_2O$): calcd. 284.1325, found 284.1322. (2*S*,5*S*)-**15d:** 139 mg, 43%. – R_f (*c*-Hex/EtOAc 1:1) = 0.10. – $[\alpha]_D^{20}$ = −117 (*c* = 1.12, $CHCl_3$). – ^{1}H NMR (400 MHz, $CDCl_3$): δ/ppm = 1.82 (bs, 1 H, N*H*), 2.53 (s, 3 H, CH_3), 3.11 (dd, ^2J = 14.0 Hz, ^3J = 4.7 Hz, 1 H, CH_2), 3.26 (dd, ^2J = 14.0 Hz, ^3J = 5.6 Hz, 1 H, CH_2), 3.86–3.88 (m,

1 H, CH_2CH), 5.12 (d, ^4J = 1.2 Hz, 1 H, NC*H*N), 8.76–8.79 (m, 2 H, 2 × H_{ar}), 8.91–8.95 (m, 2 H, 2 × H_{ar}), 7.21–7.24 (m, 2 H, 2 × H_{ar}), 7.28–7.33 (m, 3 H, 3 × H_{ar}). – ^{13}C NMR (100 MHz, $CDCl_3$): δ/ppm = 27.2 (p, NCH_3), 36.9 (s, CH_2), 60.3 (t, CH_2CH), 76.8 (t, N*C*HN), 115.9 (t, dubl, ^2J = 21.7 Hz, $C_{ar'}$-3, $C_{ar'}$-5), 126.9 (t, C_{ar}H), 128.8 (t, 2 × C_{ar}H), 128.9 (t, dubl, ^3J = 8.9 Hz, $C_{ar'}$-2, $C_{ar'}$-6), 129.8 (t, 2 × C_{ar}H), 134.4 (q, dubl, ^4J = 2.5 Hz, $C_{ar'}$-1), 136.7 (q, C_{ar}), 163.2 (q, dubl, ^1J = 249 Hz, C_{ar}-F), 174.2 (q, *C*=O). – ^{19}F NMR (376 MHz, $CDCl_3$): δ/ppm = −111.8 (s, *F*). – IR (DRIFT): $\tilde{\nu}$/cm^{-1} = 3330 (w), 3062 (w), 2930 (w), 2879 (m), 1926 (w), 1695 (s), 1604 (m), 1509 (m), 1496 (m), 1443 (m), 1400 (m), 1364 (m), 1314 (m), 1299 (m), 1263 (m), 1218 (m), 1163 (m), 1098 (m), 1076 (m), 1002 (m), 936 (w), 923 (w), 875 (m), 793 (m), 763 (w), 742 (m), 702 (m), 641 (w), 604 (m), 566 (m), 553 (m), 504 (m), 485 (w), 432 (w), 422 (w), 412 (w). – EI-MS *m/z* (%): 284 (9) [M^+], 193 (100) [$M^+-C_7H_7$]. – EI-HRMS ($C_{17}H_{17}FN_2O$): calcd. 284.1325, found 284.1328.

(2*R*,5*S*)-5-Benzyl-2-(4-bromophenyl)-3-methylimidazolidin-4-one ((2*R*,5*S*)-15e) and (2*S*,5*S*)-5-benzyl-2-(4-bromophenyl)-3-methylimidazolidin-4-one ((2*S*,5*S*)-15e):

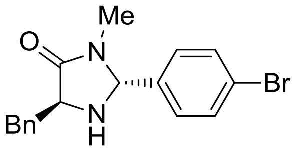

(2*R*,5*S*)-**15e**

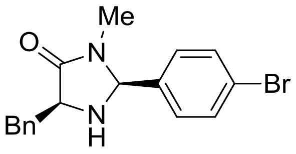

(2*S*,5*S*)-**15e**

Scale: 1.12 mmol. (2*R*,5*S*)-**15e:** 151 mg, 39%. – R_f (*c*-Hex/EtOAc 3:1) = 0.10. – $[\alpha]_D^{20}$ = 18.5 (*c* = 2.05, $CHCl_3$). – ^{1}H NMR (600 MHz, $CDCl_3$): δ/ppm = 2.10 (bs, 1 H, N*H*), 2.56 (s, 3 H, CH_3), 2.97 (dd, ^2J = 13.7 Hz, ^3J = 7.2 Hz, 1 H, CH_2), 3.12 (dd, ^2J = 13.7 Hz, ^3J = 4.1 Hz, 1 H, CH_2), 4.03–4.06 (m, 1 H, CH_2CH), 4.81 (d, ^4J = 1.7 Hz, 1 H, NC*H*N), 7.08–7.12 (m, 2 H, 2 × H_{ar}), 7.22–7.33 (m, 5 H, 5 × H_{ar}), 7.47–7.51 (m, 2 H, 2 × H_{ar}). – ^{13}C NMR (100 MHz, $CDCl_3$): δ/ppm = 27.1 (p, NCH_3), 38.6 (s, CH_2), 60.0 (t, CH_2CH), 77.1 (t, N*C*HN), 123.3 (q, C_{ar}-Br), 126.8 (t, C_{ar}H), 128.4 (t, 2 × C_{ar}H), 128.5 (t, 2 × C_{ar}H), 129.6 (t, 2 × C_{ar}H), 132.3 (t, 2 × C_{ar}H), 137.4 (q, C_{ar}), 138.4 (q, C_{ar}), 174.1 (q, *C*=O). – IR (KBr): $\tilde{\nu}$/cm^{-1} = 3468 (w), 3323 (w), 3084 (w), 3060 (w), 3028 (w), 2921 (w), 1701 (m), 1591 (w), 1484 (m), 1453 (m), 1430 (m), 1399 (m), 1355 (w), 1275 (w), 1092 (w), 1072 (w), 1030 (w), 1010 (w), 824 (w), 750 (w), 487 (w). – EI-MS *m/z* (%): 346/344 (10/9) [M^+], 255/253 (100/57) [$M^+-C_7H_7$], 200/198 (20/30). – EI-HRMS ($C_{17}H_{17}BrN_2O$): calcd. 344.0524, found 344.0522. (2*S*,5*S*)-**15e:** 174 mg, 45%. – R_f (*c*-Hex/EtOAc 3:1) = 0.03. – $[\alpha]_D^{20}$ = −87.9 (*c* = 0.97, $CHCl_3$). – ^{1}H NMR (600 MHz, $CDCl_3$): δ/ppm = 1.86 (bs, 1 H, N*H*), 2.53 (s, 3 H, CH_3),

3.09 (dd, ^2J = 14.0 Hz, ^3J = 4.7 Hz, 1 H, C*H*$_2$), 3.26 (dd, ^2J = 14.0 Hz, ^3J = 5.5 Hz, 1 H, C*H*$_2$), 3.86–3.89 (m, 1 H, CH$_2$C*H*), 5.10 (d, ^4J = 1.1 Hz, 1 H, NC*H*N), 6.64–6.67 (m, 2 H, 2 × H_{ar}), 7.19–7.23 (m, 2 H, 2 × H_{ar}), 7.29–7.33 (m, 3 H, 3 × H_{ar}), 7.36–7.38 (m, 2 H, 2 × H_{ar}). – ^{13}C NMR (100 MHz, CDCl$_3$): δ/ppm = 27.2 (p, N*C*H$_3$), 36.8 (s, *C*H$_2$), 60.2 (t, CH$_2$*C*H), 76.7 (t, N*C*HN), 123.5 (q, C_{ar}-Br), 127.0 (t, C_{ar}H), 128.8 (t, 2 × C_{ar}H), 128.9 (t, 2 × C_{ar}H), 129.9 (t, 2 × C_{ar}H), 132.1 (t, 2 × C_{ar}H), 136.6 (q, C_{ar}), 137.6 (q, C_{ar}), 174.1 (q, *C*=O). – IR (KBr): ṽ/cm^{-1} = 3471 (w), 3333 (w), 3085 (w), 3061 (w), 3028 (w), 2921 (w), 2854 (w), 1702 (m), 1591 (w), 1546 (w), 1483 (w), 1425 (w), 1398 (w), 1335 (w), 1294 (w), 1274 (w), 1095 (w), 1073 (w), 1010 (w), 822 (w), 741 (w), 701 (w), 606 (w), 488 (w), 405 (w). – EI-MS *m/z* (%): 346/344 (9/12) [M$^+$], 255/253 (98/100) [M$^+$−C$_7$H$_7$], 200/198 (17/30), 91 (30) [C$_7$H$_7$$^+$]. – EI-HRMS (C$_{17}H_{17}BrN_2$O): calcd. 344.0524, found 344.0527.

(2*R*,5*S*)-5-Benzyl-3-methyl-2-(4-cyanophenyl)imidazolidin-4-one ((2*R*,5*S*)-15f) and (2*S*,5*S*)-5-benzyl-3-methyl-2-(4-cyanophenyl)imidazolidin-4-one ((2*S*,5*S*)-15f):

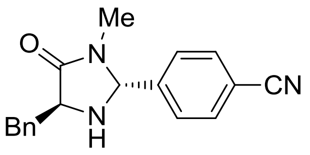

(2*R*,5*S*)-**15f**

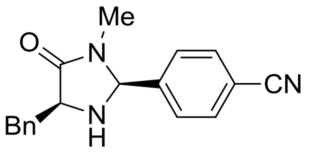

(2*S*,5*S*)-**15f**

Scale: 1.12 mmol. (2*R*,5*S*)-**15f:** 124 mg, 38%. – R_f (*c*-Hex/EtOAc 1:1) = 0.14. – $[\alpha]_D^{20}$ = 27.6 (*c* = 0.99, CHCl$_3$). – ^{1}H NMR (600 MHz, CDCl$_3$): δ/ppm = 2.16 (bs, 1 H, N*H*), 2.58 (s, 3 H, C*H*$_3$), 3.00 (dd, ^2J = 13.9 Hz, ^3J = 7.0 Hz, 1 H, C*H*$_2$), 3.12 (dd, ^2J = 13.9 Hz, ^3J = 4.2 Hz, 1 H, C*H*$_2$), 4.02–4.05 (m, 1 H, CH$_2$C*H*), 4.89 (d, ^4J = 1.0 Hz, 1 H, NC*H*N), 7.24–7.32 (m, 5 H, 5 × H_{ar}), 7.34–7.36 (m, 2 H, 2 × H_{ar}). 7.66–7.68 (m, 2 H, 2 × H_{ar}). – ^{13}C NMR (100 MHz, CDCl$_3$): δ/ppm = 27.2 (p, N*C*H$_3$), 38.5 (s, *C*H$_2$), 60.0 (t, CH$_2$*C*H), 76.8 (t, N*C*HN), 113.2 (q, C_{ar}-CN), 118.1 (q, *C*N), 126.9 (t, C_{ar}H), 127.5 (t, 2 × C_{ar}H), 128.5 (t, 2 × C_{ar}H), 129.5 (t, 2 × C_{ar}H), 132.9 (t, 2 × C_{ar}H), 137.0 (q, C_{ar}), 144.5 (q, C_{ar}), 174.0 (q, *C*=O). – IR (KBr): ṽ/cm^{-1} = 3328 (w), 3086 (w), 3061 (w), 3029 (w), 3003 (w), 2922 (m), 2855 (w), 2229 (m), 1689 (m), 1607 (w), 1496 (m), 1478 (m), 1430 (m), 1400 (m), 1366 (w), 1330 (w), 1276 (w), 1204 (w), 1095 (w), 1030 (w), 1018 (w), 999 (w), 843 (w), 754 (w), 702 (w), 566 (w), 528 (w). – EI-MS *m/z* (%): 291 (7) [M$^+$], 200 (100) [M$^+$−C$_7$H$_7$], 145 (22), 58 (23), 43 (68) [C$_2$H$_3$O$^+$]. – EI-HRMS (C$_{18}$H$_{17}$N$_3$O): calcd. 291.1372, found 291.1368. (2*S*,5*S*)-**15f:** 149 mg, 46%. – R_f (*c*-Hex/EtOAc 1:1) = 0.05. – $[\alpha]_D^{20}$ = −144 (*c* = 1.04, CHCl$_3$). – ^{1}H NMR (600 MHz, CDCl$_3$): δ/ppm = 2.12 (bs, 1 H, N*H*), 2.53 (s, 3 H, C*H*$_3$), 2.95 (dd,

^2J = 13.8 Hz, ^3J = 7.0 Hz, 1 H, CH_2), 3.08 (dd, ^2J = 13.8 Hz, ^3J = 4.2 Hz, 1 H, CH_2), 3.95–4.07 (m, 1 H, CH_2CH), 4.84 (d, ^4J = 1.6 Hz, 1 H, NC*H*N), 7.20–7.26 (m, 5 H, 5 × H_{ar}), 7.29–7.32 (m, 2 H, 2 × H_{ar}). 7.60–7.64 (m, 2 H, 2 × H_{ar}). – ^{13}C NMR (100 MHz, $CDCl_3$): δ/ppm = 27.3 (p, NCH_3), 37.1 (s, CH_2), 60.1 (t, CH_2CH), 76.6 (t, N*C*HN), 113.3 (q, C_{ar}-CN), 118.1 (q, *C*N), 126.9 (t, C_{ar}H), 128.0 (t, 2 × C_{ar}H), 128.8 (t, 2 × C_{ar}H), 129.9 (t, 2 × C_{ar}H), 132.7 (t, 2 × C_{ar}H), 136.6 (q, C_{ar}), 144.0 (q, C_{ar}), 173.9 (q, *C*=O). – IR (KBr): ṽ/cm^{-1} = 3347 (w), 3055 (w), 2967 (w), 2933 (w), 2837 (w), 2225 (w), 1951 (w), 1828 (w), 1681 (m), 1608 (w), 1487 (w), 1451 (w), 1338 (w), 1264 (w), 1205 (w), 1153 (w), 1099 (w), 1074 (w), 1005 (w), 936 (w), 908 (w), 848 (w), 811 (w), 780 (w), 450 (w), 731 (w), 698 (w), 645 (w), 616 (w), 573 (w), 533 (w), 511 (w), 482 (w), 453 (w), 433 (w), 411 (w). – EI-MS *m/z* (%): 291 (8) [M^+], 200 (100) [$M^+-C_7H_7$], 145 (20). – EI-HRMS ($C_{18}H_{17}N_3O$): calcd. 219.1372, found 291.1374.

(S,E)-*N*-methyl-2-((4-nitrobenzylidene)amino)-3-phenylpropanamide (16):

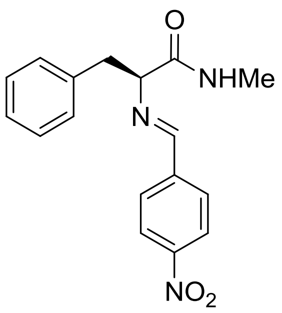

(*S*)-Phenylalanine-*N*-methylamide **13** (714 mg, 4.01 mmol, 1.00 equiv), 4-nitrobenzaldehyde (618 mg, 4.01 mmol, 1.00 equiv), $FeCl_3$ (130 mg, 801 µmol, 0.20 equiv) and molecular sieves (3 Å) were suspended in THF (10 mL) and stirred at room temperature for 3 d. The reaction mixture was filtered and diluted with 10% Na_2CO_3-solution, extracted with dichloromethane (4 × 20 mL) and dried over Na_2SO_4, and the solvent was evaporated.

823 mg, 66%. R_f = 0.40 (*n*-Hex/EtOAc 1:1 + 5% triethylamine). – mp: 150–152 °C. – $[\alpha]_D^{20}$ = 0.87 (*c* = 1.03, $CHCl_3$). – ^{1}H NMR (400 MHz, $CDCl_3$): δ/ppm = 2.90 (d, ^3J = 4.4 Hz, 3 H, CH_3), 2.97 (dd, ^2J = 13.5 Hz, ^3J = 10.1 Hz, 1 H, CH_2), 3.47 (dd, ^2J = 13.5 Hz, ^3J = 3.1 Hz, 1 H, CH_2), 4.02 (dd, ^3J = 10.1 Hz, ^3J = 3.1 Hz, 1 H, C*H*-CH_2), 6.82–6.87 (m, 1 H, N*H*), 7.05 (d, ^4J = 1.4 Hz, 1 H, 2-H_{ar}), 7.07 (d, ^4J = 1.9 Hz, 1 H, 6-H_{ar}), 7.14–7.23 (m, 3 H, 3-H_{ar}, 4-H_{ar}, 5-H_{ar}), 7.57 (s, 1 H, C*H*=N), 7.75 (d, ^3J = 8.8 Hz, 2 H, 2-$H_{ar'}$, 6-$H_{ar'}$), 8.24 (d, ^3J = 8.8 Hz, 2 H, 3-$H_{ar'}$, 5-$H_{ar'}$). – ^{13}C NMR (100 MHz, $CDCl_3$): δ/ppm = 26.0 (p, CH_3), 41.1 (s, CH_2), 75.4 (t, *C*H-CH_2), 123.9 (t, $C_{ar'}$-3, $C_{ar'}$-5), 126.7 (t, C_{ar}-4), 128.3 (t, C_{ar}-2, C_{ar}-6), 128.8 (t, C_{ar}-3, C_{ar}-5), 130.0 (t, $C_{ar'}$-2, $C_{ar'}$-6), 137.1 (q, C_{ar}-1), 140.5 (q, $C_{ar'}$-1), 149.3 (q, $C_{ar'}$-4), 160.3 (t, *C*H=N), 172.1 (q, *C*=O). – IR (DRIFT): ṽ/cm^{-1} = 3237 (w), 3078 (w), 2950 (w), 1825 (w), 2882 (w), 1652 (m), 1600 (m), 1571 (m), 1520 (s), 1494 (w), 1454 (w), 1415 (w), 1384 (w),

1351 (s), 1291 (w), 1233 (w), 1149 (w), 1107 (w), 4087 (w), 4043 (w), 1005 (w), 987 (w), 929 (w), 852 (m), 832 (w), 775 (w), 753 (w), 735 (w), 704 (m), 691 (w), 673 (w), 585 (w), 524 (w), 507 (w), 490 (w). – EI-MS (70 eV) *m/z* (%): 311 (10) [M^+], 253 (100) [$C_{16}H_{17}N_2O^+$], 220 (33) [$M^+{-}C_7H_7$], 163 (99) [$C_{10}H_{12}NO^+$], 91 (37) [$C_7H_7^+$]. – EI-HRMS ($C_{17}H_{17}N_3O_3$): calcd. 311.1268, found 311.1270. – $C_{17}H_{17}N_3O_3$ (311.1): calcd. C 65.58, H 5.50, N 13.50, found. C 65.01, H 5.28, N 13.77.

5-Methoxy-7-pentyl-3-vinyl-2*H*-chromene (17):

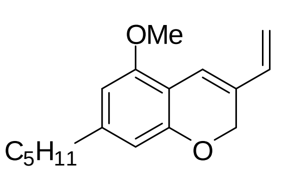

Methyltriphenylphosphonium bromide (1.68 g, 4.61 mmol, 2.00 equiv) in THF (45 mL) was cooled to −78 °C and *n*-BuLi (1.48 mL, 3.69 mmol, 1.60 equiv, 2.5 M in *n*-hexane) was added slowly. It was stirred for 30 min at −78 °C before it was warmed to 0 °C. After being stirred for 30 min the suspension of the Wittig reagent was cooled again to −78 °C and 5-methoxy-7-pentyl-2*H*-chromene-3-carbaldehyde (600 mg, 2.30 mmol, 1.00 equiv) in THF (6 mL) was added. The suspension was stirred for 30 min at −78 °C and 30 min at room temperature. After that, ethanol (1.5 mL) was added, the solvent was removed, and the product was purified by flash column chromatography (*c*-Hex/EtOAc 50:1).

533 mg, 90%. R_f = 0.40 (*c*-Hex/EtOAc 50:1). – ^{1}H NMR (400 MHz, $CDCl_3$): δ/ppm = 0.89 (t, ^3J = 7.0 Hz, 3 H, CH_3), 1.31–1.34 (m, 4 H, CH_2), 1.58–1.61 (m, 2 H, CH_2), 2.52 (t, ^3J = 7.7 Hz, 2 H, CH_2), 3.83 (s, 3 H, OCH_3), 4.89 (s, 2 H, O-CH_2), 5.05 (d, $^3J_{trans}$ = 17.8 Hz, 1 H, CH=C$H_{2,trans}$), 5.10 (d, $^3J_{cis}$ = 11.0 Hz, 1 H, CH=C$H_{2,cis}$), 6.26 (s, 1 H, H_{ar}), 6.33 (s, 1 H, H_{ar}), 6.47 (dd, $^3J_{trans}$ = 17.8 Hz, $^3J_{cis}$ = 11.0 Hz, 1 H, CH=C$H_{2,trans}$), 6.70 (s, 1 H, CH). – ^{13}C NMR (100 MHz, $CDCl_3$): δ/ppm = 14.0 (p, CH$_3$), 22.5 (s, CH$_2$), 30.8 (s, CH$_2$), 31.5 (s, CH$_2$), 36.85 (s, CH$_2$), 55.6 (p, OCH$_3$), 64.8 (s, O-CH$_2$), 103.9 (t, C_{ar}H), 108.4 (t, C_{ar}H), 109.6 (q, C_{ar}-CH), 112.0 (s, CH=CH$_2$), 118.7 (t, C_{ar}-CH), 128.1 (q, C-CH), 135.2 (t, CH=CH$_2$), 145.0 (q, C_{ar}-CH$_2$), 154.4 (q, C_{ar}-O), 155.4 (q, C_{ar}-OCH$_3$). – IR (KBr): $\tilde{\nu}$/cm^{-1} = 2955 (m), 2930 (m), 2855 (w), 1630 (w), 1612 (m), 1600 (m), 1568 (m), 1462 (m), 1428 (w), 1390 (w), 1361 (w), 1288 (w), 1227 (w), 1190 (m), 1144 (w), 1111 (m), 1056 (w), 1024 (w), 985 (w), 944 (w), 884 (w), 820 (w). – EI-MS *m/z* (%): 259 (17) [M^++H], 258 (100) [M^+], 202 (23), 201 (25) [$C_{13}H_{13}O_2^+$], 69 (21), 43 (57) [$C_3H_7^+$]. – EI-HRMS ($C_{17}H_{22}O_2$): calcd. 258.1620, found 258.1617. – $C_{17}H_{22}O_2$ (258.2): calcd. C 79.03, H 8.58, found C 79.16, H 8.20.

Ethyl 10-formyl-1-methoxy-3-pentyl-8,9,10,10a-tetrahydro-6*H*-benzo[*c*]chromene-9-carboxylate (19):

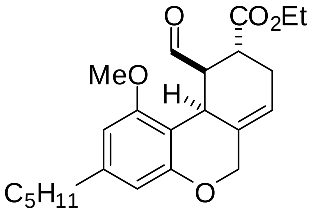

(*E*)-Ethyl-4-oxobut-2-enoate (**18**, 25.8 mg, 194 µmol, 1.00 equiv) was added to a solution of 1,3-bis(3,5-bis-(trifluoromethyl)phenyl)thiourea (19.4 mg, 38.7 µmol, 0.20 equiv) in CH_2Cl_2 (1 mL). It was stirred for 5 min, before 5-methoxy-7-pentyl-3-vinyl-2*H*-chromene (**17**, 50.0 mg, 194 µmol, 1.00 equiv), dissolved in methanol (1 mL), was added. The resulting mixture was stirred for 2 d at rt. For workup, the reaction mixture was diluted with Et_2O (2 mL), and the organic phase was washed with brine (5 mL) and dried over sodium sulfate. The solvent was removed under reduced pressure and the product was purified by flash column chromatography (*c*-Hex/EtOAc 30:1).

30.4 mg, 41%. R_f (*c*-Hex/EtOAc 30:1) = 0.09. – ^{1}H NMR (400 MHz, $CDCl_3$): δ/ppm = 0.89 (t, ^3J = 6.9 Hz, 3 H, CH_2-CH_2-CH_3), 1.29–1.36 (m, 3 H, CH_2), 1.33 (t, ^3J = 7.1 Hz, 3 H, O-CH_2-CH_3), 1.56–1.64 (m, 3 H, CH_2), 2.24–2.33 (m, 1 H, 8-*H*), 2.53 (t, ^3J = 7.7 Hz, 2 H, CH_2-CH_2-C_{ar}), 2.57–2.65 (m, 1 H, 8-*H*), 3.41 (dd, ^3J = 7.5 Hz, ^4J = 2.7 Hz, 1 H, 9-*H*), 3.80–3.82 (m, 1 H, 10a-*H*), 3.83 (s, 3 H, OCH_3), 3.89 (dd, ^3J = 4.8 Hz, ^4J = 2.7 Hz, 1 H, 10-*H*), 4.13–4.21 (m, 1 H, 6-*H*), 4.29–4.34 (m, 1 H, 6-*H*), 4.33 (q, ^3J = 7.1 Hz, 2 H, O-CH_2-CH_3), 5.75–5.78 (m, 1 H, 7-*H*), 6.33 (d, ^4J = 1.3 Hz, 1 H, H_{ar}), 6.38 (d, ^4J = 1.3 Hz, 1 H, H_{ar}), 9.19 (s, 1 H, C*H*O). – ^{13}C NMR (100 MHz, $CDCl_3$): δ/ppm = 14.0 (p, CH_2-CH_2-CH_3), 14.3 (p, O-CH_2-CH_3), 22.5 (s, CH_2), 22.7 (s, *C*-8), 30.1 (t, *C*-10a), 30.8 (s, CH_2), 31.5 (s, CH_2), 36.0 (s, CH_2), 38.4 (t, *C*-9), 48.3 (t, *C*-10), 55.3 (p, OCH_3), 60.9 (s, O-CH_2), 70.4 (s, *C*-6), 103.9 (t, *C*-4), 108.3 (q, *C*-10b), 110.1 (t, *C*-2), 125.2 (t, *C*-7), 132.0 (q, *C*-6a), 143.7 (q, *C*-3), 157.8 (q, *C*-4a), 157.9 (q, *C*-1), 174.5 (q, $CO_2CH_2CH_3$), 202.8 (t, *C*HO). – IR (KBr): $\tilde{\nu}$/cm^{-1} = 2931 (m), 2856 (w), 1719 (s), 1616 (m), 1578 (m), 1459 (m), 1425 (m), 1376 (w), 1329 (w), 1289 (w), 1198 (m), 1167 (m), 1109 (m), 1037 (m), 925 (w), 828 (w). – EI-MS *m/z* (%): 387 (24) [M^++H], 386 (100) [M^+], 313 (65) [$M^+-CO_2C_2H_5$], 283 (45) [$M^+-CO_2C_2H_5-OCH_3$]. – EI-HRMS ($C_{23}H_{30}O_5$): calcd. 386.2093, found 386.2091.

2,2-Dimethyl-3-vinyl-2*H*-chromene (20):

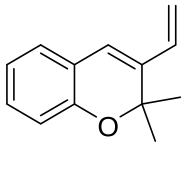

Methyltriphenylphosphoniumbromide (3.15 g, 8.65 mmol, 2.00 equiv) in THF (40 mL) was cooled to −78 °C and *n*-BuLi (1.48 mL, 3.69 mmol, 1.60 equiv, 2.5 M in *n*-hexane) was added

slowly. It was stirred for 30 min at −78 °C before it was warmed to 0 °C. After being stirred for 30 min the suspension of the Wittig reagent was cooled again to −78 °C and 2,2-dimethyl-2*H*-chromene-3-carbaldehyde (970 mg, 4.33 mmol, 1.00 equiv) in THF (10 mL) was added. The suspension was stirred for 30 min at −78 °C and 30 min at room temperature. After that, ethanol (5 mL) was added, the solvent was removed, and the product was purified by flash column chromatography (*c*-Hex/EtOAc 50:1).

858 mg, 80%. R_f = 0.37 (*n*-Hex/EtOAc 50:1). – ^{1}H NMR (250 MHz, $CDCl_3$): δ/ppm = 1.41 (s, 6 H, 2 × CH_3), 5.06 (dd, $^3J_{cis}$ = 11.0 Hz, ^5J = 1.5 Hz, 1 H, CH-C$H_{2,\,cis}$), 5.46 (dd, $^3J_{trans}$ = 17.3 Hz, ^5J = 1.5 Hz, 1 H, CH-C$H_{2,\,trans}$), 6.18 (ddd, $^3J_{trans}$ = 17.3 Hz, $^3J_{cis}$ = 11.0 Hz, ^4J = 1.0 Hz, 1 H, C*H*-CH_2), 6.37 (s, 1 H, C*H*), 6.70 (d, ^3J = 7.9 Hz, 1 H, 5-H_{ar}), 6.78 (dd, ^3J = 7.5 Hz, ^4J = 1.2 Hz, 1 H, 7-H_{ar}), 6.92 (dd, ^3J = 7.5 Hz, ^4J = 1.8 Hz, 1 H, 8-H_{ar}), 7.00 (dd, ^3J = 7.9 Hz, ^4J = 1.8 Hz, 1 H, 6-H_{ar}).

Ethyl 10-formyl-6,6-dimethyl-8,9,10,10a-tetrahydro-6*H*-benzo[*c*]chromene-9-carboxylate (21)

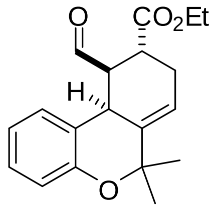

(*E*)-Ethyl-4-oxobut-2-enoate (**18**, 2.26 mL, 2.41 g, 18.8 mmol, 1.00 equiv) was added to a solution of diene **20** (3.50 g, 18.8 mmol, 1.00 equiv) in CH_2Cl_2 (60 mL) and the solution was stirred overnight at rt. After evaporation of the solvent, the crude product was purified by flash column chromatography (*c*-Hex/EtOAc 30:1).

R_f (*c*-Hex/EtOAc 5:1) = 0.40. – ^{1}H NMR (400 MHz, $CDCl_3$): δ/ppm = 1.24 (s, 3 H, 6-CH_3), 1.25 (t, ^3J = 7.1 Hz, 3 H, CO_2CH_2C*H_3*), 1.49 (s, 3 H, 6-CH_3), 2.21 (m_c, 1 H, 8-H_A), 2.58 (ddd, ^2J = 19.2 Hz, $^3J_{8,9}$ = 3.1 Hz, $^3J_{8,7}$ = 3.1 Hz, 1 H, 8-H_B), 3.29 (dd, $^3J_{9,8}$ = 7.1 Hz, $^3J_{9,10}$ = 2.9 Hz, 1 H, 9-H), 3.45 (dd, $^3J_{10,10a}$ = 4.9 Hz, $^3J_{10,9}$ = 2.9 Hz, 1 H, 10-H), 3.76 (m_c, 1 H, 10a-H), 4.18 (m_c, 2 H, CO_2C*H_2*CH_3), 5.70 (dd, $^3J_{7,8A}$ = 7.1 Hz, $^3J_{7,8B}$ = 3.1 Hz, 1 H, 7-H), 6.78 (dd, $^3J_{4,3}$ = 8.1 Hz, $^4J_{4,2}$ = 1.1 Hz, 1 H, 4-H), 6.89 (dd, $^3J_{2,1}$ = 7.6 Hz, $^3J_{2,3}$ = 7.4 Hz, 1 H, 2-H), 7.09 (ddd, $^3J_{3,4}$ = 8.1 Hz, $^3J_{3,2}$ = 7.4 Hz, $^4J_{3,1}$ = 1.5 Hz, 1 H, 3-H), 7.16 (d, $^3J_{1,2}$ = 7.6 Hz, 1 H, 1-H), 9.07 (s, 1 H, C*H*O). – ^{13}C NMR (100 MHz, $CDCl_3$): δ = 14.3 (p, CH_2CH_3), 22.9 (s, C-8), 25.6 (p, 6-*C*H_3), 25.9 (p, 6-*C*H_3), 30.7 (t, C-10a), 37.9 (t, C-9), 51.0 (t, C-10), 61.1 (s, *C*H_2CH_3), 76.2 (q, C-6), 118.1 (t, C-4), 120.0 (t, C-7), 121.2 (t, C-2), 122.0 (q, C-10b), 127.3 (t, C-3), 128.1 (t, C-1), 137.0 (q, C-6a), 154.0 (q, C-4a), 173.9 (q, *C*O_2Et), 202.5 (t, *C*HO). – IR (KBr): ṽ/cm^{-1} = 3062 (w), 2981 (m), 2934 (m), 2907 (m), 2836 (m), 2737

(w), 1722 (s), 1608 (w), 1584 (m), 1487 (s), 1455 (s), 1384 (m), 1367 (m), 1340 (w), 1305 (m), 1261 (s), 1239 (s), 1190 (s), 1140 (s), 1094 (m), 1052 (m), 1034 (m), 939 (m), 912 (w), 890 (w), 862 (w), 817 (w), 757 (s), 702 (w), 527 (w). – EI-MS (70 eV): *m/z* (%) = 315 (4) $[M^{+}+H]$, 314 (19) $[M^{+}]$, 300 (17) $[M^{+}+H-CH_3]$, 299 (49) $[M^{+}-CH_3]$, 226 (16), 225 (100), 197 (15), 43 (19). – EI-HRMS ($C_{12}H_{12}O_2$): calcd. 314.1518, found 314.1517. – $C_{19}H_{22}O_4$ (314.2): calcd. C 72.09, H 7.05, found C 72.42, H 7.09.

We also tested imidazolidinone catalysts **15** on a model system **20** without the pentyl chain, but bearing two geminal methyl groups, also typical for the THC scaffolds.

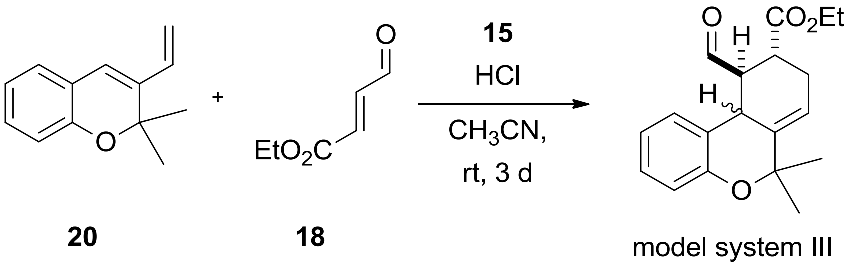

Scheme S1: Catalyst screening on model system III.

Table S1: Summarized results of catalyst screening on model system **20**.

Entry	Catalyst		*cis*-**21** Yield [%]	*cis*-**21** ee [%]
1	*t*-Bu	(2*R*,5*S*)-**15a**	19	−41
2		(2*S*,5*S*)-**15a**	14	−73
3	*o*-NO_2-C_6H_4	(2*R*,5*S*)-**15b**	21	n.d.
4		(2*S*,5*S*)-**15b**	n.d.	−13
5	*m*-F-C_6H_4	(2*R*,5*S*)-**15c**	22	−7
6		(2*S*,5*S*)-**15c**	27	−4
7	*p*-F-C_6H_4	(2*R*,5*S*)-**15d**	18	−31
8		(2*S*,5*S*)-**15d**	27	−4
9	*p*-CN-C_6H_4	(2*R*,5*S*)-**15e**	16	−53
10		(2*S*,5*S*)-**15e**	21	−4
11	$(CH_3)_2$	(5*R*)-**15g**	30	+59

References

1. Yan, Z.; Cai, X.; Yang, X.; Song, B.; Chen, Z.; Bhadury, S. P.; Hu, D.; Jin, L.; Xue, W.; Lu, P. *Chin. J. Chem.* **2009,** *27,* 593–601. doi:10.1002/cjoc.200990097
2. Hernández-Rodríguez, M.; Avila-Ortiz, C. G.; del Campo, J. M.; Hernández-Romero, D.; Rosales-Hoz, M. J.; Juaristi, E. *Aust. J. Chem.* **2008,** *61,* 364–375. doi:10.1071/CH08116
3. Breuzard, J. A. J.; Tommasino, M. L.; Touchard, F.; Lemaire, M.; Bonnet, M. C. *J. Mol. Catal. A: Chem.* **2000,** *156,* 223–232. doi:10.1016/S1381-1169(99)00401-X
4. Sibi, M. P.; Itoh, K. *J. Am. Chem. Soc.* **2007,** *129,* 8064–8065. doi:10.1021/ja071739c
5. MacMillan, D. W. C. Enantioselective transformations of α,β-unsaturated aldehydes by a wide variety of reactions using imidazolidinone enantiomers as chiral organic catalysts. Patent WO 2003002491, January, 9, 2003.
